# Guizhi-Shaoyao-Zhimu decoction attenuates rheumatoid arthritis partially by reversing inflammation-immune system imbalance

**DOI:** 10.1186/s12967-016-0921-x

**Published:** 2016-06-08

**Authors:** Qiuyan Guo, Xia Mao, Yanqiong Zhang, Shuqin Meng, Yue Xi, Yi Ding, Xiaocun Zhang, Yuntao Dai, Xia Liu, Chao Wang, Yuting Li, Na Lin

**Affiliations:** Institute of Chinese Materia Medica, China Academy of Chinese Medical Sciences, Beijing, 100700 China; Department of Pathology, Beijing Jishuitan Hospital, Peking University, Beijing, 100035 China; School of Chemistry, Chemical Engineering and Life Science, Wuhan University of Technology, Hubei, 430070 China

**Keywords:** TCM herbal formula, Rheumatoid arthritis, Guizhi-Shaoyao-Zhimu decoction, Network pharmacology, Experimental validation

## Abstract

**Background:**

Guizhi-Shaoyao-Zhimu decoction (GSZD) has been extensively used for rheumatoid arthritis (RA) therapy. Marked therapeutic efficacy of GSZD acting on RA has been demonstrated in several long-term clinical trials without any significant side effects. However, its pharmacological mechanisms remain unclear due to a lack of appropriate scientific methodology.

**Methods:**

GSZD’s mechanisms of action were investigated using an integrative approach that combined drug target prediction, network analysis, and experimental validation.

**Results:**

A total of 77 putative targets were identified for 165 assessed chemical components of GSZD. After calculating the topological features of the nodes and edges in the created drug-target network, we identified a candidate GSZD-targeted signal axis that contained interactions between two putative GSZD targets [histone deacetylase 1 (HDAC1) and heat shock protein 90 kDa alpha, class A member 1 (HSP90AA1)] and three known RA-related targets [NFKB2; inhibitor of kappa light polypeptide gene enhancer in B-cells, kinase beta (IKBKB); and tumor necrosis factor-alpha (TNF-α)]. This signal axis could connect different functional modules that are significantly associated with various RA-related signaling pathways, including T/B cell receptor, Toll-like receptor, NF-kappa B and TNF pathways, as well as osteoclast differentiation. Furthermore, the therapeutic effects and putative molecular mechanisms of GSZD’s actions on RA were experimentally validated in vitro and in vivo.

**Conclusions:**

GSZD may partially attenuate RA by reversing inflammation-immune system imbalance and regulating the HDAC1–HSP90AA1–NFKB2–IKBKB–TNF-α signaling axis.

**Electronic supplementary material:**

The online version of this article (doi:10.1186/s12967-016-0921-x) contains supplementary material, which is available to authorized users.

## Background

Rheumatoid arthritis (RA) is a chronic, debilitating inflammatory joint disease characterized by synovial inflammation and the progressive destruction of cartilage and bone [1]. Several studies have indicated that RA has a prevalence rate of approximately 0.5 to 1 % in adult populations in developed countries [2]. Moreover, growing evidence indicates that RA patients are more at risk of developing a collection of comorbidities that have no typical features and are difficult to diagnose, leading them to have poorer clinical outcomes than that of the general population [3]. Current therapeutic agents, such as non-steroidal anti-inflammatory drugs (NSAIDs), disease-modifying anti-rheumatic drugs (DMARDs), glucocorticoids, and biological response modifiers, have been used to reduce inflammation, relieve pain, suppress disease activity, prevent joint damage, and slow the progression of RA [4]. However, their poor efficacies, high prices and adverse effects are of concern [5, 6]. An increasing number of patients with RA worldwide are seeking help from complementary and alternative medicine to alleviate the severity of the disease and to improve physical conditions. Among these treatments, traditional Chinese medicine (TCM) is regarded as a powerful treatment option, and it has been used for RA therapy for thousands of years in China [7].

In TCM, RA is categorized as “arthromyodynia” (Bi Zheng, Bi syndrome or blockage syndrome) [8, 9]. Various TCM-based herbal formulae and extracts have been reported to effectively relieve the severity of RA. Among them, Guizhi-Shaoyao-Zhimu Decoction (GSZD), as a classic TCM-based herbal formula originally recorded by the famous Chinese physician Zhang Zhongjing in “*Synopsis of the prescriptions of the golden chamber”* (Chinese name: Jin Gui Yao Lue) is widely produced in China in accordance with China Pharmacopoeia standards of quality control. GSZD is composed of nine Chinese herbs, including Ramulus Cinnamomi (Guizhi), *Paeonia lactiflora (Shaoyao)*, Radix Glycytthizae (Gancao), Herba Ephedrae (Mahuang), Rhizoma Zingiberis Recens (Shengjiang), Rhizoma Atractylodis Macrocephalae (Baizhu), Rhizoma Anemarrhenae (Zhimu), Raidix Saposhnikoviae (Fangfeng) and Radix Aconiti Lateralis Preparata (Fuzi). Recent clinical studies have revealed that the clinical cure rates of GZSD on treatment of patients with RA may range from 87.5 to 95.8 %, superior to those of indometacin, tripterygium glycosides and prednisone [10–13]. In addition to its marked efficacy, no significant side effects of GZSD have been observed in several long-term trials in China [14]. Modern medical research has shown that GSZD can alleviate RA progression by restraining osteoclast differentiation and activation, reducing synovial cell proliferation, and increasing synovial cell apoptosis both in vivo and in vitro [15, 16]. However, the underlying mechanisms of this formula’s actions on RA have not been fully clarified.

**Fig. 1:**
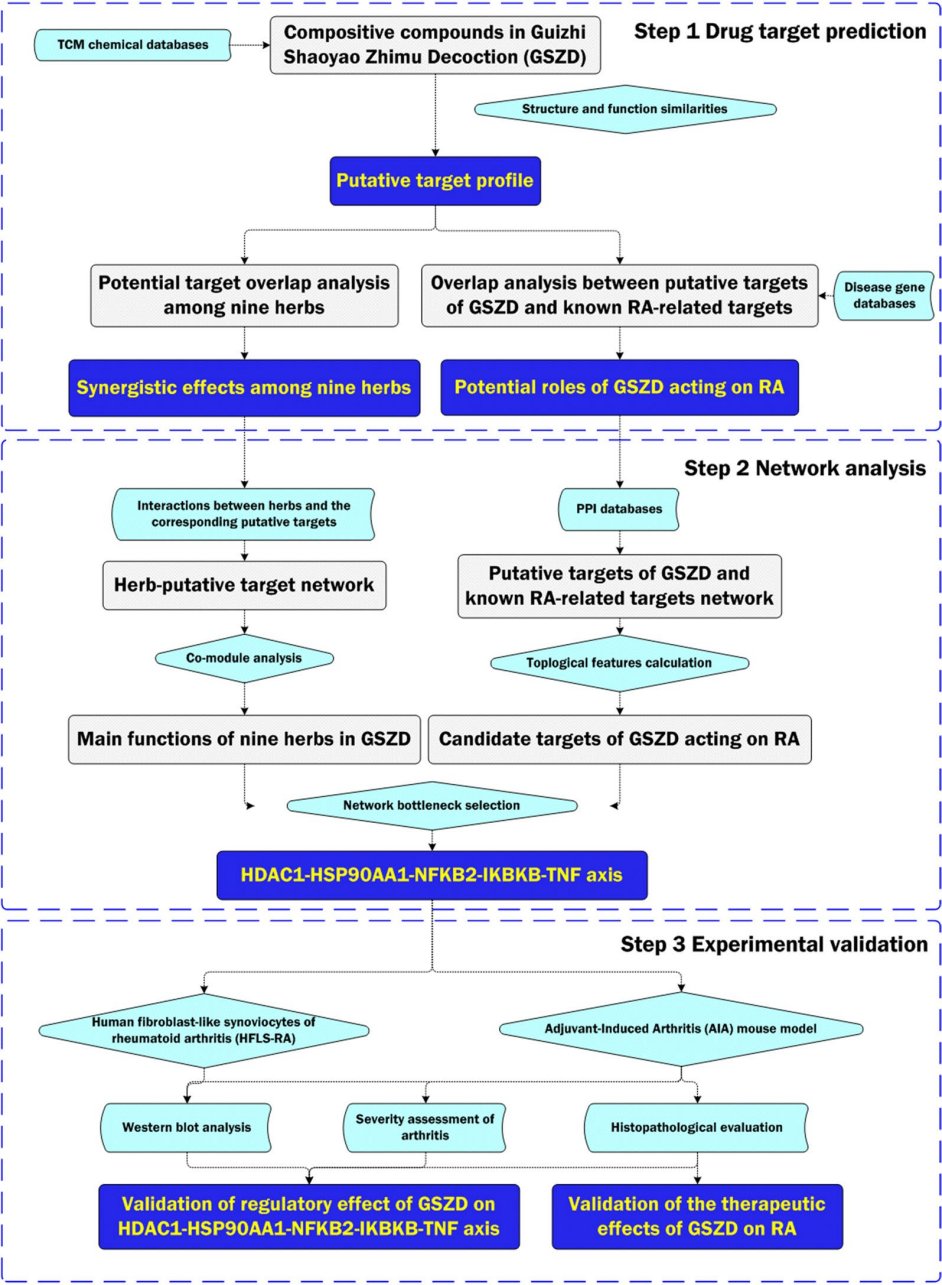
A schematic diagram of the systematic strategies for unraveling the pharmacological mechanisms of herbal formula GSZD acting on RA

TCM formulae containing large numbers of composite compounds are too complex to be assessed by traditional experimental methods based on the “one gene, one drug, one disease” paradigm [17]. Growing evidence shows that the synergistic and holistic philosophy underlying the creation of TCM formulae is consistent with the main view of the emerging concept of network pharmacology, which is based on the rapid progress of systems biology, network biology and poly-pharmacology [18]. By applying a set of network-based methods, network pharmacology can define TCM from a systems perspective and at a molecular level, providing a new method of translating TCM from an experience-based to an evidence-based medical system by integrating network-based computational predictions and experimental validations [19, 20]. In the current study, as shown in Fig. 1, we predicted the putative targets of GSZD based on drug structures and functions, constructed and analyzed the herb-target network and the putative target-RA related gene network, and performed in vitro and in vivo experimental validations to highlight that the therapeutic effects of GSZD on RA might be associated with its roles in reversing the imbalance of inflammation-immune system during the disease progression.

## Methods

### Data preparation

#### Composite compounds of each herb in GSZD

The composite compounds of each herb in GSZD were obtained from TCM Database@Taiwan (http://tcm.cmu.edu.tw/, updated in 2012-06-28), which is currently the largest non-commercial TCM database worldwide [21]. Detailed information on the composite compounds of each herb in GSZD is provided in Additional file 1: Table S1.

#### Known RA-related targets

Known RA-related targets were collected from existing resources, including the DrugBank database [22] (http://www.drugbank.ca/, version 3.0), the Online Mendelian Inheritance in Man (OMIM) database [23] (http://www.omim.org/, last updated on October 31, 2013), the Genetic Association Database (GAD) [24] (http://geneticassociationdb.nih.gov/, last updated on August 18, 2013) and the Kyoto Encyclopedia of Genes and Genomes (KEGG) Pathway Database [25] (http://www.genome.jp/kegg/, last updated on October 16, 2012), in accordance with our previous study. Detailed information on these known therapeutic targets is provided in Additional file 1: File S1-Sect. 1 and Table S2.

#### Molecular interaction data

The interaction data corresponding to the putative targets of GSZD and known RA-related targets were collected from existing databases [26–33] (Additional file 1: Table S3).

### Prediction of putative GSZD targets

As described in our previous studies [34, 35], the Drug Similarity Search tool in the Therapeutic Targets Database (TTD, http://xin.cz3.nus.edu.sg/group/cjttd/ttd.asp, Version 4.3.02 released on Aug 25th 2011) was used to identify drugs similar to the herbs contained in GSZD via structural similarity comparison. We only selected drugs with high similarity scores (>0.85, similar–very similar) to the structures of the composite compounds of the herbs contained in GSZD. The therapeutic targets of the similar drugs were identified as putative targets of the herbs contained in GSZD. The performance of this prediction method has been assessed in our previous studies [34, 35].

### Network construction and analysis

The putative herb-target network, putative targets of GSZD and known RA-related targets network and hub-pathway network were constructed and visualized using Navigator software (Version 2.2.1).

To assess the topological properties of each node in the networks, five features, including ‘Degree’, ‘Closeness’, ‘Node-betweenness’, ‘K-coreness’ and ‘Edge-betweenness’, were calculated according to our previous studies [34–36]. Please see the detailed information in Additional file 1: File S1-Sect. 2.

### Experimental validation

The study was approved by the Research Ethics Committee of the Institute of Chinese Materia Medica, China Academy of Chinese Medical Sciences, Beijing, China. All procedures conformed to the guidelines and regulations for the use and care of animals of the Center for Laboratory Animal Care, China Academy of Chinese Medical Sciences.

#### Preparation of GSZD

Based on the composition of GSZD recorded in the Chinese Pharmacopoeia 2010 edition, GSZD was prepared using the following procedure. Radix Aconiti Lateralis Preparata (10 g) and a mixture of Ramulus Cinnamomi (12 g), *Paeonia lactiflora* (9 g), Rhizoma Anemarrhenae (12 g), Rhizoma Atractylodis Macrocephalae (15 g), Radix Saposhnikoviae (12 g), Herba Ephedrae (12 g), Radix Glycytthizae (6 g), and Rhizoma Zingiberis Recens (15 g) were separately soaked with pure water for 30 min. Then, Radix Aconiti Lateralis Preparata was added to 1040 mL (1:10 g/v) of boiling pure water and boiled for 30 min; the herb mixture was then added and boiled for 40 min. The filtrates were collected, and the residues were decocted in 520 mL (1:5 g/v) of water for 40 min. The filtrates from each decoction were combined and concentrated to 1 g/mL at 90 °C. The obtained GSZD was stored at 4 °C prior to use. High-performance liquid chromatography with diode array detection (HPLC–DAD) fingerprinting was used to quantify the main chemical components of the nine herbs contained in GSZD.

#### Animals

Experiments were performed on 6-week-old male Lewis rats at a weight of 180–220 g, which were purchased from Beijing Vital River Laboratory Animal Technology Ltd (production license No: SCXK 2012-0001). All animals were housed in a temperature-controlled room at a constant temperature of 24 ± 1 °C (mean ± SD) with a 12-h light/dark cycle. Food pellets and water were provided ad libitum.

#### Cell culture and drug treatment

HFLS-RA (Cell Applications, USA) cells were used for in vitro experimental validation. The cells were cultured in sterile synoviocyte growth medium (Cell Applications, USA) supplemented with 100 U/mL 1 penicillin, 80 U/mL 1 streptomycin, and 2 mM glutamine and were maintained at 37 °C in a humidified atmosphere of 5 % CO_2_/95 % air. The cells were used between passage 4 and 8 and were incubated with 10 ng/mL IL-1β and different concentrations of GSZD (5.12 × 10^−5^, 2.56 × 10^−4^ and 1.28 × 10^−3^ μg/mL) for 24 h.

#### Induction and treatment of AIA in rats

Arthritis was induced in rats by inoculation with Freund’s complete adjuvant (CFA). Briefly, the rats were injected intradermally at the base of the tail with 0.1 mL CFA (1 mg of heat-killed *Mycobacterium tuberculosis* suspended in 0.1 mL paraffin oil; Difco). Control group rats were injected with an equal volume of saline instead of CFA. With this protocol, the first signs of inflammation were observed on day 11 after adjuvant injection.

GSZD treatment began on the day of CFA immunization and was administered daily for a period of 21 days. A GSZD solution was prepared at a concentration of 0.9 g/mL and delivered by oral administration. Male Lewis rats were divided into six groups: a normal control group (Normal, n = 12), an AIA model control group (Model, n = 20), GSZD-low/middle/high groups (n = 8 per group) and a 0.2 mg/kg MTX group (MTX, n = 8). The dosage selections for the low-, middle- and high-GSZD groups were nearly equivalent to 0.5, 1 and 2 times the daily RA patient dosage. The AIA rats were treated with 4.65 g/(kg day) GSZD (GSZD-low), 9.3 g/(kg day) GSZD (GSZD-middle) and 18.6 g/(kg day) GSZD (GSZD-high). Detailed information on water and food consumption and the body weight changes in each group throughout the 21 day experiment period is provided in Additional file 1: Tables S4–S6.

#### Assessment of arthritis severity

Arthritis severity was evaluated as in our previous study [36]. Please see the detailed information provided in Additional file 1: File S1—Sect. 3.

#### Histological observation

Histology was evaluated as in our previous study [36]. Please see the detailed information provided in Additional file 1: File S1–Sect. 4.

#### Western blot analysis

Western blotting and semi-quantitative analysis were performed as in our previous study [36]. To investigate the effect of GSZD on the expression levels of HDAC1, HSP90AA1, NFKB2, IKBKB and TNF-α proteins, HFLS were treated with 10 ng/mL of IL-1β in the presence of various concentrations of GSZD. Antibodies against the following proteins were used: HDAC1 (rabbit polyclonal antibody; dilution 1:1000; Abcam, Cambridge, UK), HSP90AA1 (rabbit polyclonal antibody; dilution 1:500; Abcam, Cambridge, UK), NFKB2 (rabbit monoclonal antibody; dilution 1:1000; Cell Signaling), IKKB (rabbit monoclonal antibody; dilution 1:1000; Abcam, Cambridge, UK), and TNF-α (rabbit polyclonal antibody; dilution 1:100; Abcam, Cambridge, UK). All experiments were performed in triplicate. The mean normalized protein expression ± SD was calculated from independent experiments.

#### Statistical analysis

Statistical analyses were performed using SPSS version 13.0 for Windows (SPSS Inc, Chicago, IL, USA). Continuous variables were expressed as $\overline{X} \pm s .$ Arthritis incidence and the percentage of arthritic limbs were analyzed by Chi square tests. The arthritis index and the pathological score were analyzed with non-parametric statistics (Kruskal–Wallis test). Other data were analyzed by one-way ANOVA followed by Fisher’s LSD test. P values less than 0.05 were considered statistically significant.

## Results and discussion

### Inference of RA-related pathological processes affected by GSZD

Because drug indications are often determined by the functions of their corresponding targets and drugs with similar chemical structures generally exert similar therapeutic effects, we predicted the putative targets of nine herbs contained in GSZD based on the similarities in drug structure and function as described by our previous studies [34, 35]. A total of 77 putative targets were identified out of 165 chemical components containing in GSZD (Additional file 1: Table S7).

Then, we compared known drugs with similar structures to the chemical components in the nine GSZD herbs. As a result, the nine GSZD herbs shared 15 putative targets with known drugs for the treatment of RA, autoimmune diseases, inflammatory diseases, and pain, as well as for the provision of anesthesia (Table 1).

**Table 1:** Information of similar known drugs and the corresponding putative targets of GSZD

Herbs	Known drug	Putative target of GSZD	Type of target	Indication
Raidix Saposhnikoviae	Propofol	FAAH	Successful target	Anesthetic
Radix Glycytthizae	Fluocinonide	PLA2G1B	Successful target	Inflammatory diseases
Herba Ephedrae/Radix Glycytthizae/Paeonia lactiflora/Rhizoma Zingiberis Recens	Amcinonide/betamethasone/fluorometholone/fluticasone/medrysone/methylprednisolone/prednisone	NR3C1	Successful target	Inflammatory diseases/Rheumatoid arthritis
Herba Ephedrae/Radix Glycytthizae/Paeonia lactiflora/Rhizoma Zingiberis Recens	Alclometasone/fluocinolone acetonide/fluocinonide/flurandrenolide/paramethasone/prednisolone/triamcinolone	SERPINA6	Successful target	Autoimmune diseases/Inflammatory diseases/Rheumatoid arthritis
Herba Ephedrae/Radix Aconiti Lateralis Preparata/Radix Glycytthizae/Raidix Saposhnikoviae/Ramulus Cinnamomi/Rhizoma Atractylodis Macrocephalae/Rhizoma Zingiberis Recens/	Glycopyrrolate	CHRM1	Successful target	Anesthetic
Herba Ephedrae/Radix Aconiti Lateralis Preparata/Radix Glycytthizae/Raidix Saposhnikoviae/Ramulus Cinnamomi/Rhizoma Atractylodis Macrocephalae/Rhizoma Zingiberis Recens	Butalbital/ethanol/methohexital/talbutal/thiamylal	GABRA1	Successful target	Anesthetic/Pain/Sedation
Rhizoma Zingiberis Recens	Frovatriptan/sumatriptan/rizatriptan/almogran	HTR1D	Successful target	Pain
Rhizoma Zingiberis Recens	Almotriptan/eletriptan/frovatriptan/naratriptan/zolmitriptan	HTR1B	Successful target	Pain
Radix Glycytthizae	Hydrocortisone	NOS2	Successful target	Inflammatory diseases
Raidix Saposhnikoviae/Rhizoma Zingiberis Recens	Ibuprofen/etodolac/carprofen	PTGS2	Successful target	Pain
Herba Ephedrae/Radix Aconiti Lateralis Preparata/Radix Glycytthizae/Raidix Saposhnikoviae/Ramulus Cinnamomi/Rhizoma Atractylodis Macrocephalae/Rhizoma Zingiberis Recens	Naloxone	OPRM1	Successful target	Anesthetic
Radix Aconiti Lateralis Preparata/Radix Glycytthizae	Codeine/Hydromorphone	OPRD1	Successful target	Pain
Rhizoma Zingiberis Recens	Naloxone	FNTA	Successful target	Anesthetic
Radix Glycytthizae	*N*-acetyl-d-glucosamine	NAGLU	Successful target	Autoimmune diseases
Rhizoma Zingiberis Recens	Metocurine/mivacurium/pipecuronium	CHRNA2	Successful target	Anesthetic/Pain

The putative targets of Herba Ephedrae, Radix Aconiti Lateralis Preparata, Radix Glycytthizae, Raidix Saposhnikoviae, Ramulus Cinnamomi, Rhizoma Atractylodis Macrocephalae and Rhizoma Zingiberis Recens included nitric oxide synthase 2 (NOS2); nuclear receptor subfamily 3, group C, member 1 (NR3C1); phospholipase A2, group IB (PLA2G1B); and serpin peptidase inhibitor, clade A, member 6 (SERPINA6), which are the targets of several glucocorticoids and FDA-approved anti-inflammatory and immunosuppressive agents for the treatment of RA, such as Alclometasone, Amcinonide, Betamethasone, etc. based on DrugBank (Version 4.3, http://www.drugbank.ca/), suggesting that these putative targets might be involved in the anti-inflammatory and immunosuppressive effects exerted by GSZD on RA.

The management of pain is an important component of RA patient care, and cholinergic receptor, nicotinic, alpha 2 (CHRNA2), gamma-aminobutyric acid A receptor (GABRA1), 5-hydroxytryptamine receptor 1B (HTR1B), 5-hydroxytryptamine receptor 1D (HTR1D), NOS2; opioid receptor, delta 1 (OPRD1) and prostaglandin-endoperoxide synthase 2 (PTGS2) have been identified as therapeutic targets for severe pathologic pain. The current study predicted that Herba Ephedrae, Radix Aconiti Lateralis Preparata, Radix Glycytthizae, Raidix Saposhnikoviae, Ramulus Cinnamomi, Rhizoma Atractylodis Macrocephalae and Rhizoma Zingiberis Recens might target these molecules.

Providing anesthesia to patients with osteoarticular disorders during RA progression involves a number of risks not only due to the mechanical deformations caused by the disease but also in relation to the cardiovascular, respiratory, renal, and digestive systems [37]. Thus, to benefit RA patients, it is of great clinical significance to control anesthesia effectively. Here, seven GSZD herbs, including Herba Ephedrae, Radix Aconiti Lateralis Preparata, Radix Glycytthizae, Raidix Saposhnikoviae, Paeonia lactiflora, Ramulus Cinnamomi and Rhizoma Zingiberis Recens, shared targets [cholinergic receptor muscarinic 1 (CHRM1), CHRNA2, fatty acid amide hydrolase (FAAH), farnesyltransferase CAAX box alpha (FNTA), GABRA1 and OPRM1] with known anesthetic drugs, including glycopyrrolate, methohexital, metocurine, mivacurium, naloxone, propofol and thiamylal.

Collectively, the putative targets of GSZD mainly have roles in the progression of inflammation, joint destruction and pathological pain. As such, GSZD’s therapeutic efficacy in the treatment of RA may arise from its regulation of the expression or activities of these targets.

### Combinatorial effects of herbs contained in GSZD acting on RA

**Fig. 2:**
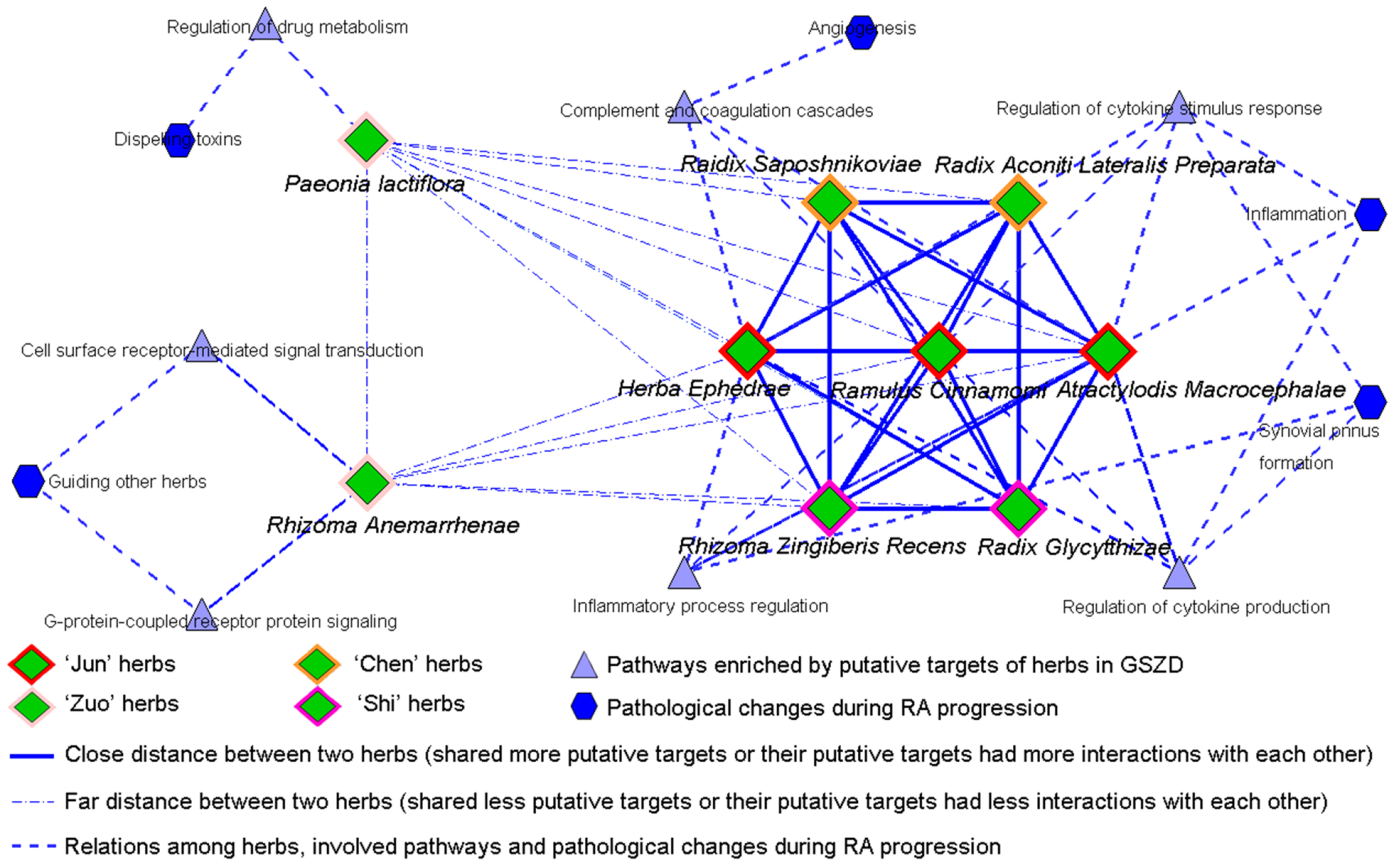
The co-module underlying GSZD formula and pathways/pathological changes involved into RA progression. Co-module analysis was performed by mapping the herbs into shared modules if the distance between two herbs was very close (shared more putative targets or their putative targets had more interactions with each other) in the network. As a result, the herb-putative target network was divided into three modules, which were centered on Ramulus Cinnamomi, *Paeonia lactiflora* and Rhizoma Anemarrhenae

The compatibility of a TCM herbal formula emphasizes the “Jun (emperor)–Chen (minister)–Zuo (adjuvant)–Shi (messenger)” rule with proper herbs to synergize the therapeutic efficacies and minimize adverse effects integrally [38, 39]. According to the co-module analysis [40], the herb-putative target network was divided into three modules, which were respectively centered on Ramulus Cinnamomi, *Paeonia lactiflora* and Rhizoma Anemarrhenae (Fig. 2). In TCM theory, Ramulus Cinnamomi, Rhizoma Atractylodis Macrocephalae and Herba Ephedrae are considered as the “Jun” herbs and play the leading roles in GSZD [41]. These herbs were linked to anti-inflammatory and anti-allergy activities in previous study [42] and were computationally confirmed here. Their putative targets were significantly associated with the regulation of inflammatory process, cytokine stimulus response and cytokine production, and complement and coagulation cascades, which are all involved into the main pathological changes during RA progression, such as inflammation, synovial pnnus formation and angiogenesis. Radix Aconiti Lateralis Preparata and Raidix Saposhnikoviae function as “Chen” herbs which enhance the pharmacological actions of the “Jun” herbs. Radix Glycytthizae and Rhizoma Zingiberis Recens are considered as “Shi” herbs and harmonize the actions of other herbs in GSZD [43]. Moreover, *Paeonia lactiflora* and Rhizoma Anemarrhenae serve as “Zuo” herbs which dispel toxins and guide other drugs to their corresponding meridian channels [44]. Similarly, we found that the biological functions and pathways of the *Paeonia lactiflora* and Rhizoma Anemarrhenae-centered modules respectively focused on the regulation of drug metabolism and cell surface receptor-mediated signal transduction, and G-protein-coupled receptor protein signaling.

### GSZD has a reverse effect on inflammation-immune regulatory network imbalance during RA progression

To elucidate the function of herb putative targets of GSZD, pathway enrichment analysis were performed and found that the top 6 pathways that the GSZD putative targets were significantly associated with neuroactive ligand-receptor interaction, toll-like receptor signaling, osteoclast differentiation, calcium signaling pathway, complement and coagulation cascades and VEGF signaling (all P < 0.001, Additional file 1: Table S8).

**Fig. 3:**
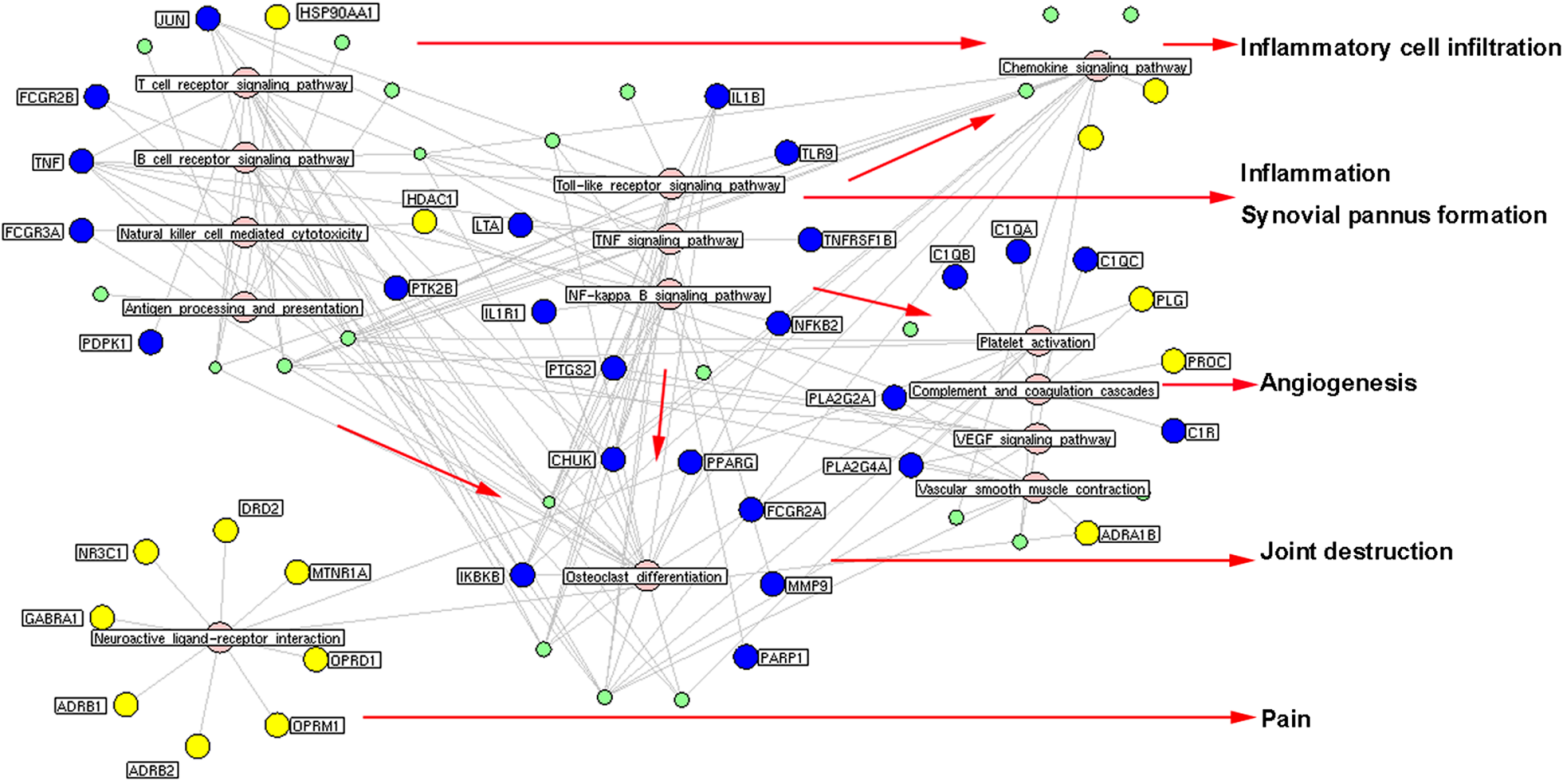
Hub-pathway network of GSZD. The pathway enrichment analysis showed that the hubs, identified from the interaction network of putative targets of GSZD and known RA-related targets, were more frequently implicated into T/B cell receptor signaling pathway, Toll-like receptor signaling pathway, Osteoclast differentiation, NF-kappa B signaling pathway, TNF signaling pathway, Chemokine signaling pathway, VEGF signaling pathway and Neuroactive ligand-receptor interaction, which all play crucial roles in the main pathological events during the RA progression, such as inflammation, synovial pannus formation, inflammatory cell infiltration, angiogenesis, joint destruction and pain. *Yellow nodes* refer to the putative targets of GSZD; *Blue nodes* refer to the known RA-related targets; *Green nodes* refer to other human genes interacted with the putative targets of GSZD or the known RA-related targets

Performing molecular network-based analysis by mapping disease-related genes and drug target genes into an interaction network can efficiently illustrate underlying links between drugs and disease. Thus, we constructed a network based on interactions between putative GSZD targets, known RA-related targets and other human proteins. A node may function as a hub if its degree is more than two-fold of the median degree of all nodes in a network [45]. As a result, 135 hubs were identified, and our pathway enrichment analysis showed that these hubs were frequently implicated in T and B cell receptor signaling, Toll-like receptor signaling, osteoclast differentiation, NF-kappa B signaling, TNF signaling, chemokine signaling, VEGF signaling, and neuroactive ligand-receptor interactions. All of these actions play crucial roles in the main pathological events that comprise RA progression, such as inflammation, synovial pannus formation, inflammatory cell infiltration, angiogenesis, joint destruction and pain [46] (Fig. 3).

Subsequently, a network of hubs based on the direct interactions of the above was constructed (please see the interaction network data in Additional file 1: Table S9). The major hubs of this network were identified by calculating four topological features of each hub in the network: ‘Degree,’ ‘Node betweenness’, ‘Closeness’ and ‘K value’. The median values of ‘Degree’, ‘Node betweenness’, ‘Closeness’ and ‘K value’ were 8.00, 0.45, 41.31 and 6.00, respectively. Therefore, we determined that hubs with ‘Degree’ >8.00, ‘Node betweenness’ >0.45, ‘Closeness’ >41.31, and ‘K value’ >6.00 were major hubs. As a result, 40 major hubs were identified (the detailed information on the topological features of the 40 major hubs is provided in Additional file 1: Table S10). After assessing the intersection of the above with the putative GSZD targets (Additional file 1: Table S7), 10 major hubs were identified as candidate targets for this formula, including albumin (ALB); androgen receptor (AR); cyclin-dependent kinase 1 (CDK1); estrogen receptor 1 (ESR1); histone deacetylase 1 (HDAC1); heat shock protein 90 kDa alpha, class A member 1 (HSP90AA1); NR3C1; retinoic acid receptor alpha (RARA); signal transducer and activator of transcription 3 (STAT3); and vitamin D receptor (VDR).

**Fig. 4:**
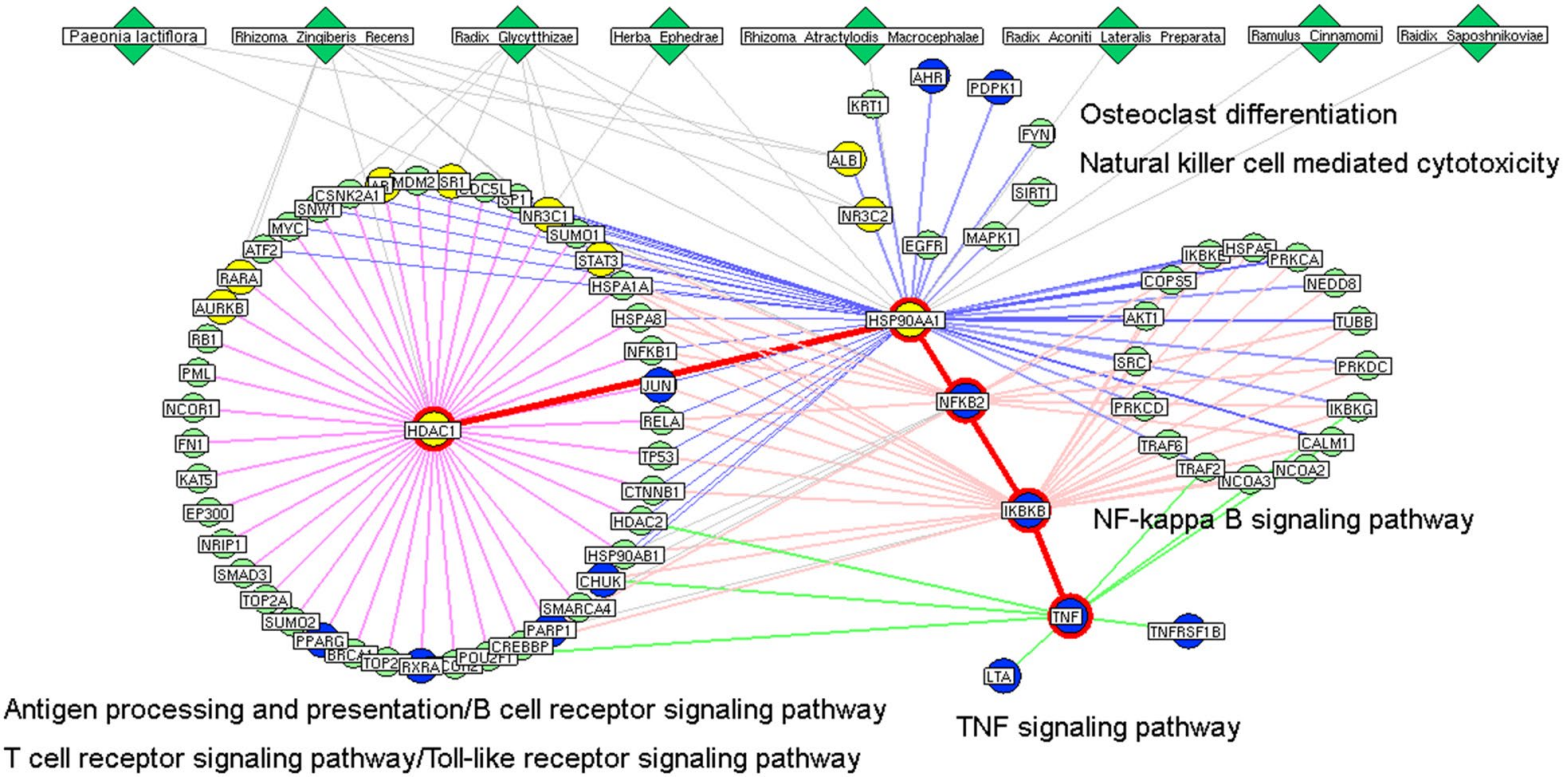
Network of interactions among herbs of GSZD and hubs obtained from the network of putative targets of GSZD and known RA-related targets.* Yellow nodes* refer to the putative targets of GSZD;* Blue nodes* refer to the known RA-related targets;* Green nodes* refer to other human genes interacted with the putative targets of GSZD or the known RA-related targets

Growing evidence has shown that an interaction with a high ‘edge-betweenness’ may function as a bottleneck with many ‘shortest paths’ going through it and may thus control the rate of information flow [47]. Here, we further calculated the ‘edge-betweenness’ of each interaction in the network of direct interactions among hubs to select important interactions. Among the candidate GSZD targets, the HDAC1–HSP90AA1 interaction had the highest edge-betweenness value (128.25, Additional file 1: Table S11), suggesting that it functions as a bottleneck in the network. As shown in the interaction network of GSZD herbs and hubs (Fig. 4), a signal axis containing interactions between HDAC1, HSP90AA1 and three known RA-related targets, including nuclear factor of kappa light polypeptide gene enhancer in B-cells 2 (NFKB2), inhibitor of kappa light polypeptide gene enhancer in B-cells, kinase beta (IKBKB) and tumor necrosis factor-alpha (TNF-α), was found to play a crucial role in connecting different modules. These modules were significantly associated with antigen processing and presentation, T and B cell receptor signaling, Toll-like receptor signaling, natural killer cell-mediated cytotoxicity, osteoclast differentiation, NF-kappa B signaling and TNF signaling, implying that GSZD might reverse the inflammation-immune regulatory network imbalance that occurs during RA progression partially by regulating the HDAC1–HSP90AA1–NFKB2–IKBKB–TNF-α axis. To validate this hypothesis, an adjuvant-induced arthritis (AIA) rat model was constructed and used to demonstrate the preventive effects of GSZD on inflammation and joint destruction. Following this, its regulatory effects on the HDAC1–HSP90AA1–NFKB2–IKBKB–TNF-α axis were also assessed both in vitro and in vivo.

### GSZD treatment ameliorates the development and severity of arthritis in AIA rats

**Fig. 5:**
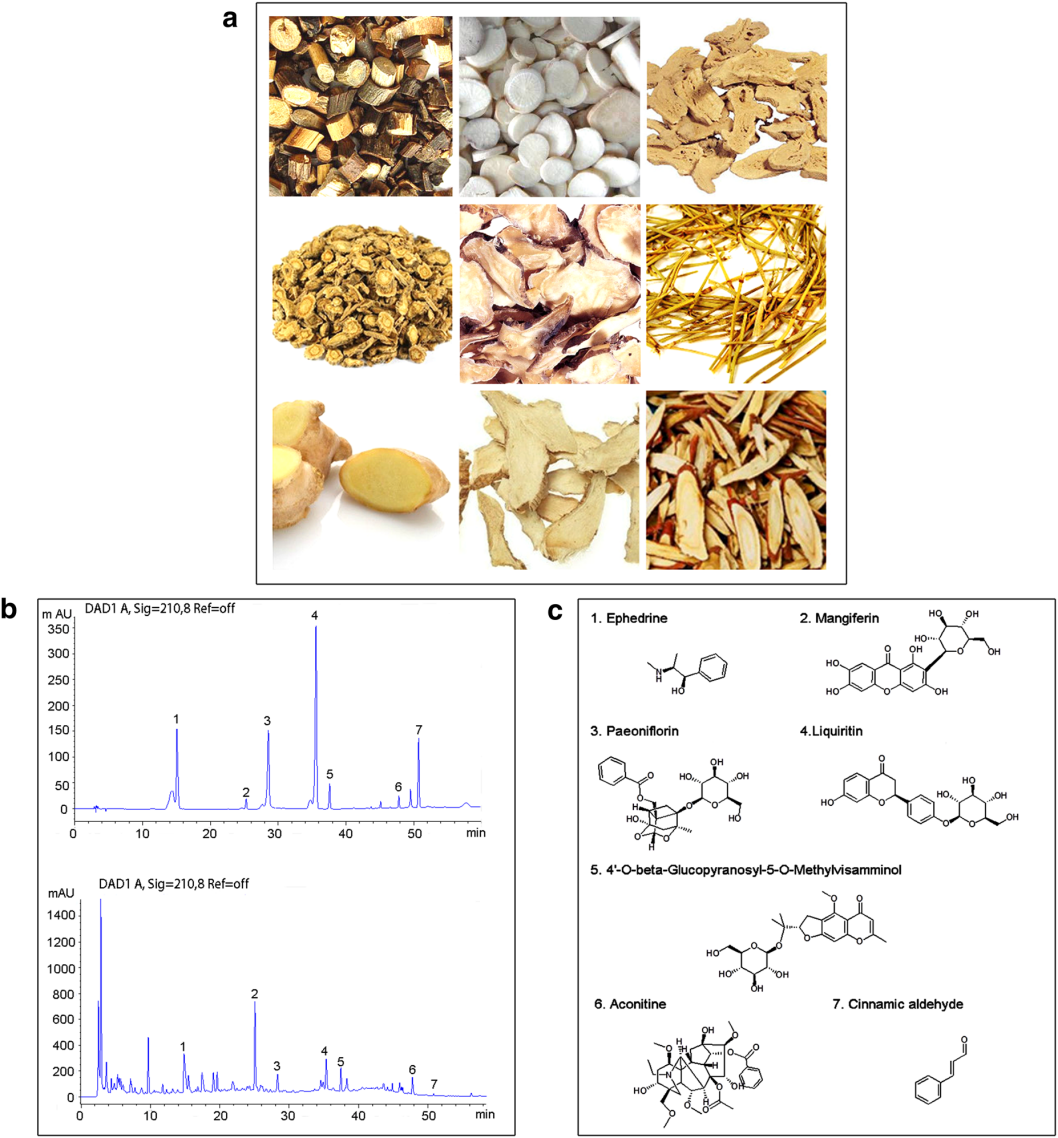
The herbal formula GSZD.** a** Photos of nine Chinese herbs in GSZD, including Ramulus Cinnamomi (Guizhi, GZ), Paeonia lactiflora (Shaoyao, SY), Rhizoma Atractylodis Macrocephalae (Baizhu, BZ), Raidix Saposhnikoviae (Fangfeng, FF), RadixAconiti Lateralis Preparata (Fuzi, FZ), Herba Ephedrae (Mahuang, MH), RhizomaZingiberis Recens (Shengjiang, SJ), Rhizoma Anemarrhenae (Zhimu, ZM) and RadixGlycytthizae (Gancao, GC) in order.** b** HPLC graphs of GSZD. HPLC was performed to identify the phytochemical profiles of GSZD.** c** The main chemicals in GSZD

Through HPLC–DAD, Ephedrine from Herba Ephedrae, mangiferin from Rhizoma Anemarrhenae, paeoniflorin from *Paeonia lactiflora*, liquiritin from Radix Glycytthizae, 4′-*O*-beta-Glucopyranosyl-5-*O*-Methylvisamminol from Raidix Saposhnikoviae, aconitine from Radix Aconiti Lateralis Preparata, and cinnamic aldehyde from Ramulus Cinnamomi were identified in the water extract of GSZD (Fig. 5).

**Fig. 6:**
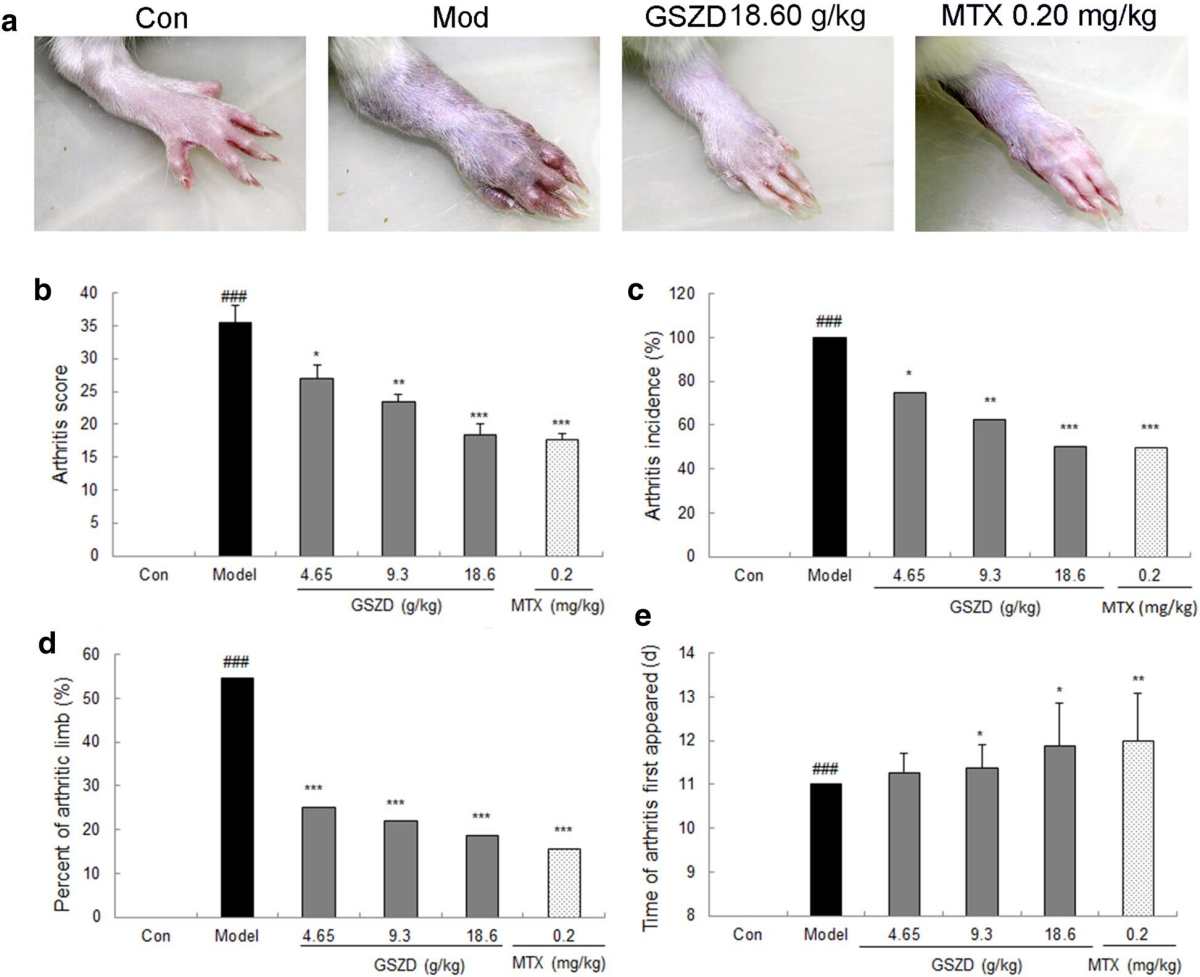
Effects of GSZD on the severity of arthritis in AIA rats. **a** Macroscopic evidence of arthritis such as redness or swelling was obviously observed in AIA rats of the model group, while doses of 18.6 g/(kg day) GSZD and 0.2 mg/(kg day) MTX significantly reduced the severity of arthritis in AIA rats; **b** Doses of 4.65–18.6 g/(kg day) GSZD and 0.2 mg/(kg day) MTX significantly reduced the mean arthritis score of AIA rats; **c** Doses of 4.65–18.6 g/(kg day) GSZD and 0.2 mg/(kg day) MTX significantly reduced the arthritis incidence of AIA rats; **d** Doses of 4.65–18.6 g/(kg day) GSZD and 0.2 mg/(kg day) MTX significantly reduced the percentage of arthritis limbs of AIA rats; **e** Doses of 9.3 g/(kg day) and 18.6 g/(kg day) GSZD, and 0.2 mg/(kg day) MTX effectively extended the time of arthritis first appeared of AIA rats. Data are represented as the mean ± SD. ^#^P < 0.05, comparison with the normal control (Con). *, **, and ***, P < 0.05, P < 0.01, and P < 0.001, respectively, comparison with the model control (Mod)

Macroscopic changes of arthritis, such as redness and swelling, were clearly observed in the AIA rats (Fig. 6a), but were attenuated by the treatment of GSZD [18.6 g/(kg day)] and MTX [0.2 mg/(kg day)]. Statistically, the mean arthritis score (all *P* < 0.05, Fig. 6b), arthritis incidence (all *P* < 0.05, Fig. 6c), percentage of arthritic limbs (all *P* < 0.05, Fig. 6d) and time of first appearance of arthritis (for doses of 9.3 and 18.6 g/(kg day), *P* < 0.05, Fig. 6e) were markedly lower in the GSZD-treated rats, especially in the middle- and high-dosage groups, and in the MTX-treated rats compared to the untreated AIA rats.

### GSZD treatment protects against synovitis and joint destruction in AIA rats

**Fig. 7:**
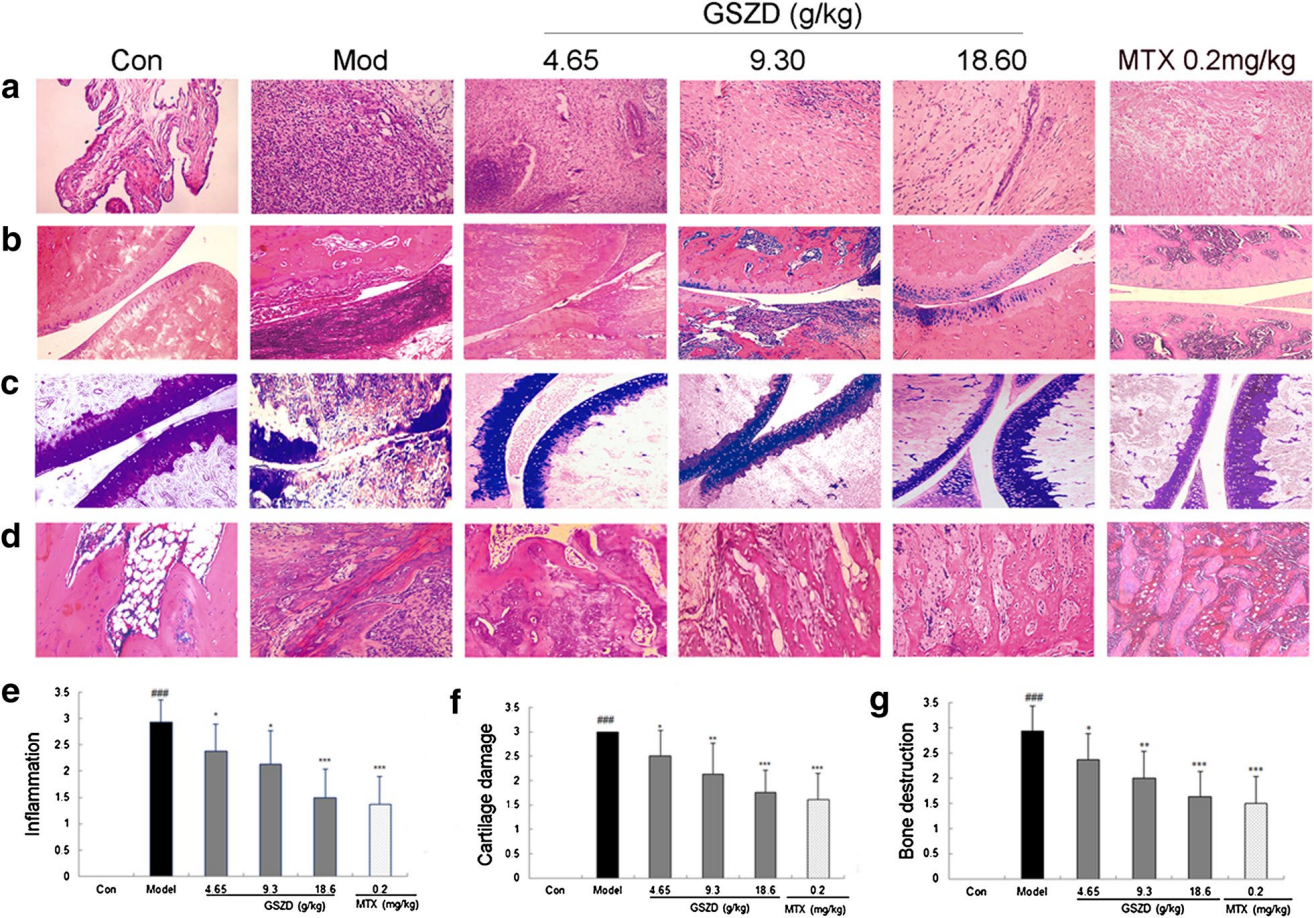
Effect of GSZD on histologic lesions of AIA rats. **a** Inflammatory changes observed in different groups using H & E staining (×200); **b** Articular cartilage changes observed in different groups using H & E staining (×200); **c** Cartilage changes observed in different groups using toluidine blue staining; (×200); **d** Bone destruction changes observed in different groups using H & E staining (×200); **e**–**g** showed the inflammation score, the bone destruction score and the loss of toluidine blue staining in joints respectively, calculated as described in “Methods” section. Data are represented as the mean ± SD. *, **, and ***, P < 0.05, P < 0.01, and P < 0.001, respectively, in contrast with the model control (Mod)

Histopathological evaluation of ankle joint sections from the AIA rats showed inflammatory cell infiltration, synovial hyperplasia and articular tissue destruction, which all could be attenuated by the oral administration of GSZD (Fig. 7). In brief, synovial edema and extensive infiltration of inflammatory cells occurred in the AIA rats, but were repaired by the treatment of GSZD, which promoted the proliferation and maturation of fibrovascular granulation tissues and reduced the number of inflammatory cells (Fig. 7a). Cartilage tissue thinning, dissolution and disappearance, as well as extensive inflammatory cell infiltration with plasma cells and lymphocytes, was observed in the articular cartilage of the ankles of the untreated AIA rats. In contrast, GSZD treatment prevented cartilage degeneration and markedly reduced inflammation by promoting cartilage cell proliferation and calcification and reducing inflammatory cell infiltration (Fig. 7b). Similarly, GSZD treatment typically preserved articular cartilage matrix integrity in markedly inflamed joints, as indicated by the retention of toluidine blue staining in the matrix (Fig. 7c). Moreover, the AIA rats showed severe bone destruction with inflammatory cell infiltration and phagocytosis of osteoclasts, which were reversed by the oral administration of GSZD mainly via the promotion of osteoblast proliferation and the acceleration of the calcification and ossification of regenerated cartilage tissues (Fig. 7d).

We statistically evaluated the anti-inflammatory and bone protective effects of GSZD with semi-quantitative grading scales (on a scale of 0–3) [48] and assessed articular cartilage matrix integrity in different groups based on the loss of toluidine blue staining [49]. As shown in Fig. 7e and f, the inflammation score and degree of cartilage damage in the GSZD-treated AIA rats were significantly decreased in a dose-dependent manner compared to the untreated AIA rats (all *P* < 0.05). Treatment with GSZD also significantly and dose-dependently reduced bone destruction in inflamed joints (all *P* < 0.05, Fig. 7g). More interestingly, the therapeutic effects produced by high-dosage GSZD treatment on inflammation score, degree of cartilage damage and bone destruction score in inflamed joints in AIA rats did not significantly differ from those produced by MTX treatment (Fig. 7).

### GSZD treatment partially reverses RA progression by targeting the HDAC1–HSP90AA1–NFKB2–IKBKB–TNF-α axis in vitro and in vivo

**Fig. 8:**
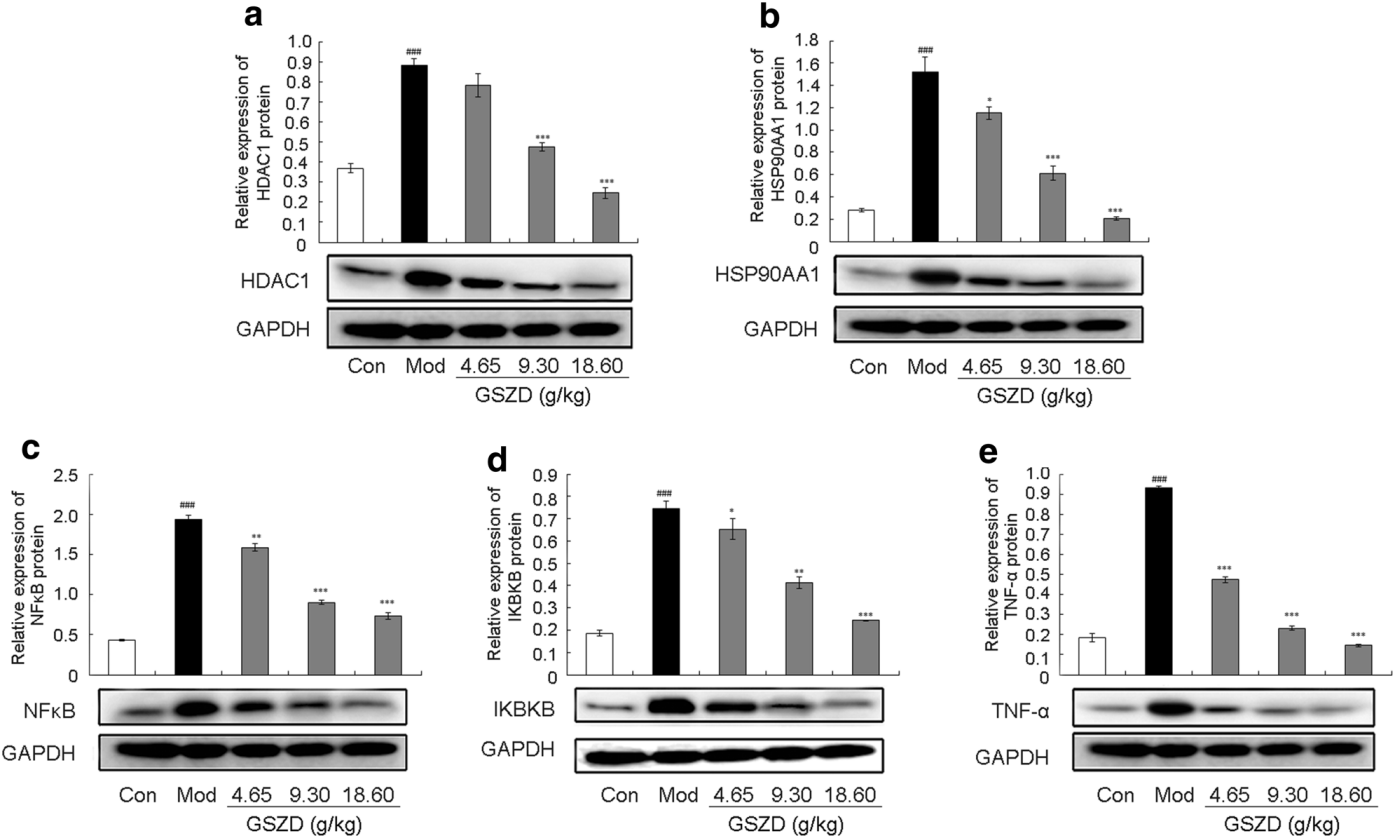
Effect of GSZD on the expression of HDAC1 (**a**), HSP90AA1 (**b**), NFKB2 (**c**), IKBKB (**d**) and TNF-α (**e**) proteins in the joint of AIA rats detected by Western blot analysis. Data are represented as the mean ± SD. ^#^ and ^##^, P < 0.05 and P < 0.01, respectively comparison with the normal control (Con). * and **, P < 0.05 and P < 0.01, respectively, comparison with the model control (Mod)

**Fig. 9:**
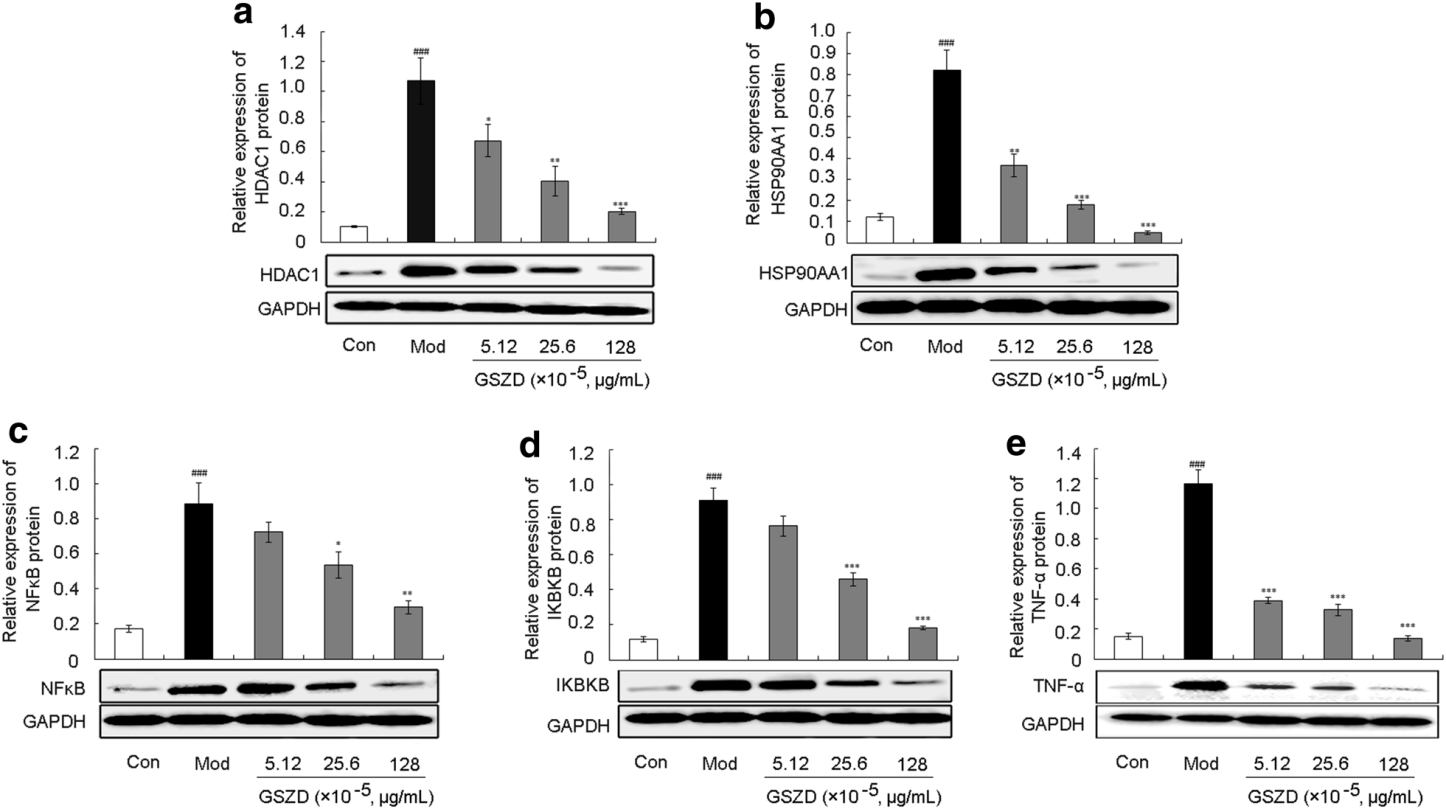
Effect of GSZD on the expression of HDAC1 (**a**), HSP90AA1 (**b**), NFKB2 (**c**), IKBKB (**d**) and TNF-α (**e**) proteins in HFLS-RA. Data are represented as the mean ± SD. ^#^ and ^##^, P < 0.05 and P < 0.01, respectively, comparison with the control cells (Con). * and **, P < 0.05 and P < 0.01, respectively, comparison with the IL-1β-induced model control (Mod)

To reveal the pharmacological mechanisms of GSZD’s action on AIA, the expression levels of HDAC1, HSP90AA1, NFKB2, IKBKB and TNF-α proteins in the inflamed joints of AIA rats and in the human fibroblast-like synoviocytes-rheumatoid arthritis (HFLS-RA) cell line were detected by western blot analysis following different treatment protocols. Compared to normal controls, HDAC1, HSP90AA1, NFKB2, IKBKB and TNF-α protein expression were markedly increased in the inflamed joints of AIA rats (all P < 0.05, Fig. 8) but were efficiently reduced by GSZD treatment. Compared with untreated AIA rats, GSZD treatment at doses of 9.3 and 18.6 g/(kg day) significantly reduced the expression of HDAC1 (all P < 0.05, Fig. 8a) and HSP90AA1 (all P < 0.05, Fig. 8b). Notably, the administration of GSZD markedly and dose-dependently decreased the expression levels of NFKB2, IKBKB and TNF-α proteins (all P < 0.05, Fig. 8c–e). More importantly, these findings were consistent with the results from in vitro experiments performed on cultured HFLS-RA, as shown in Fig. 9.

Innate immune responses in the rheumatoid synovium contribute to inflammation and joint destruction in RA [50]. NFKB2, IKBKB and TNF-α have recently been identified to play crucial roles in this chronic inflammation of synovial joint linings, which has initiated the development of a series of targeted and highly effective therapeutics for RA. Mammalian HDACs can be divided into two classes: class I HDACs (HDACs 1, 2, 3, 8), which are homologues of yeast PRD3, and class II HDACs (HDACs 4–7 and 9), which are homologues of yeast Hda1 [51]. It has been reported that HDAC1 activity and expression are dramatically increased in RA synovial tissues compared to normal tissues and are upregulated by TNF-α stimulation in RASFs, suggesting the need to develop HDAC1 inhibitors for the treatment of RA [52]. HSP90AA1, a chaperone family member, functions to guide the late-stage tertiary folding of numerous proteins [53]. HSP90AA1 guides the folding of NF-kappa B signaling pathway members, such as receptor-interacting protein and IKK, which can be degraded following HSP90AA1 inhibition, blocking NF-kappa B signaling pathway activation and causing a subsequent loss of cytokine production in macrophages and other cell types [54]. Thus, accumulating evidence suggests that an HSP90AA1-targeted agent would be useful in the treatment of inflammatory diseases, including RA. Here, we employed in vivo and in vitro experimental validation to demonstrate that GSZD ameliorates the upregulation of HDAC1, HSP90AA1, NFKB2, IKBKB, and TNF-α, in line with its role in reducing synovial inflammation and preventing cartilage destruction during RA progression.

## Conclusions

In the current study, we integrated drug target prediction and network analysis to assess the multiple ingredients and putative targets of GSZD, a TCM-based herbal formula, which enabled us to clarify its pharmacological actions on RA. Our network analysis inferred associations between candidate targets of the herbs contained in GSZD and components in the pathological processes of RA and discerned key essential mechanisms of the formula. Furthermore, in vitro and in vivo experimental validation offered convincing evidence that GSZD may partially attenuate RA by reversing inflammation-immune system imbalance and regulating the HDAC1–HSP90AA1–NFKB2–IKBKB–TNF-α signaling axis.

Although there are important discoveries revealed by this study, there are also limitations. First, some composite compounds of herbs contained in GSZD might have been omitted due to incomplete information obtained from existing databases. Second, this work could not determine whether the identified associations between the studied herbs and their corresponding targets were direct or indirect. Third, since each data source used here may have its own set of constraints, biases or limitations, etc., which might impact the final results; However, we did not adequate to define these characteristics before data integration and data mining. Thus, more studies will be required in the future.

## Additional file

10.1186/s12967-016-0921-x Integrated analysis of network pharmacology-based prediction and experimental validation reveals that Guizhi-Shaoyao-Zhimu decoction attenuates rheumatoid arthritis partially by reversing inflammation-immune system imbalance.
