# Correction to: Guizhi-Shaoyao-Zhimu decoction attenuates rheumatoid arthritis partially by reversing inflammation-immune system imbalance

**DOI:** 10.1186/s12967-020-02536-0

**Published:** 2020-09-29

**Authors:** Qiuyan Guo, Xia Mao, Yanqiong Zhang, Shuqin Meng, Yue Xi, Yi Ding, Xiaocun Zhang, Yuntao Dai, Xia Liu, Chao Wang, Yuting Li, Na Lin

**Affiliations:** 1 Institute of Chinese Materia Medica, China Academy of Chinese Medical Sciences, Beijing, 100700 China; 2 Department of Pathology, Beijing Jishuitan Hospital, Peking University, Beijing, 100035 China; 3 School of Chemistry, Chemical Engineering and Life Science, Wuhan University of Technology, Hubei, 430070 China

## Correction to: J Transl Med (2016) 14:165 10.1186/s12967-016-0921-x

Following publication of the original article [1], the authors identified an error in Fig. 7. It was brought to the authors’ attention that the cartilage photograph of the MTX group in Fig. 7c was misplaced. The correct Fig. 7 and its accompanying caption are given below.

**Fig. 7:**
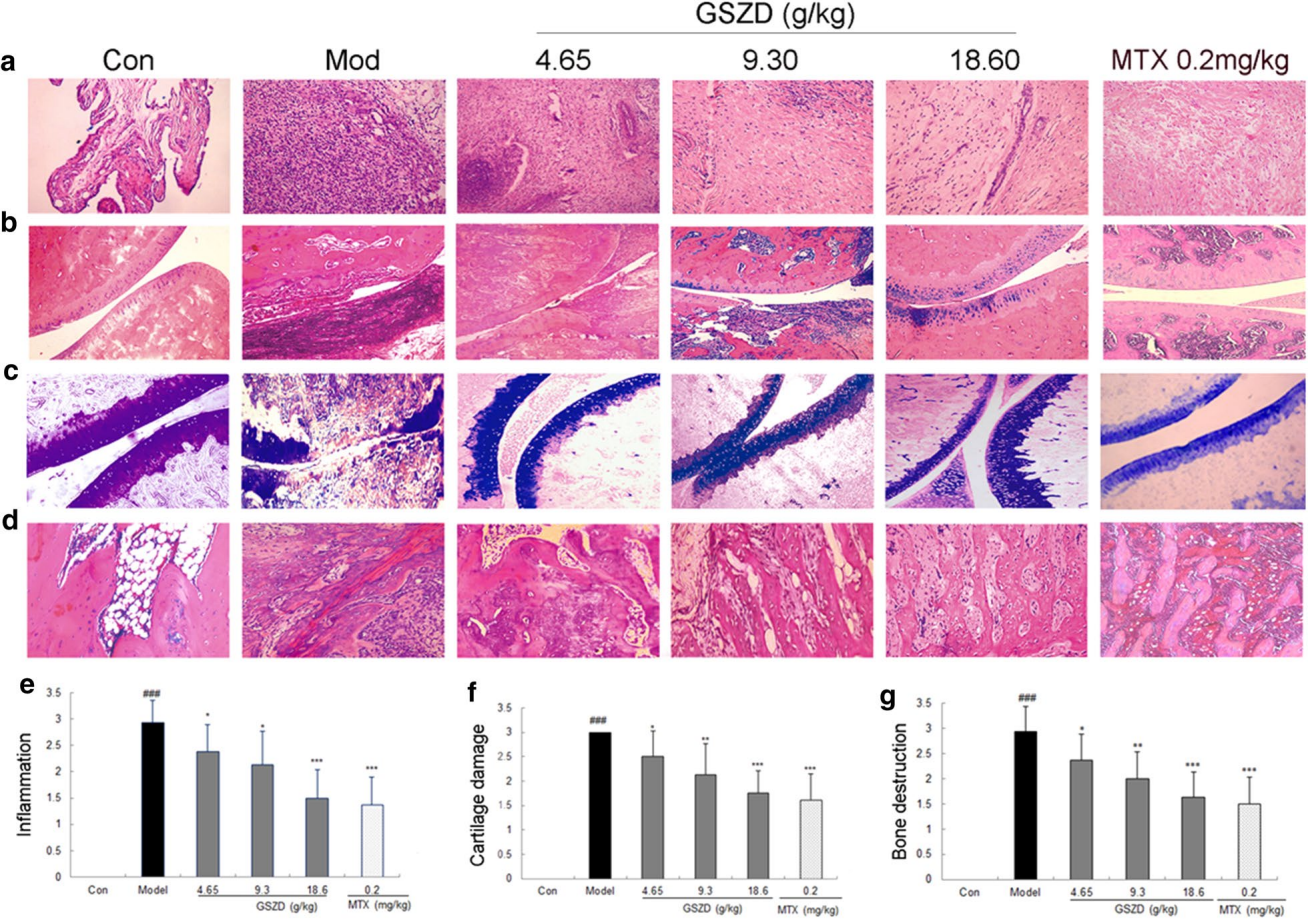
Effect of GSZD on histologic lesions of AIA rats. **a** Inflammatory changes observed in different groups using H & E staining (×200); **b** Articular cartilage changes observed in different groups using H & E staining (×200); **c** Cartilage changes observed in different groups using toluidine blue staining; (×200); **d** Bone destruction changes observed in different groups using H & E staining (×200); **e**–**g** showed the inflammation score, the bone destruction score and the loss of toluidine blue staining in joints respectively, calculated as described in “Methods” section. Data are represented as the mean ± SD. *, **, and ***, P < 0.05, P < 0.01, and P < 0.001, respectively, in contrast with the model control (Mod)
