# Supplementary material for: Guizhi-Shaoyao-Zhimu decoction attenuates rheumatoid arthritis partially by reversing inflammation-immune system imbalance
Source: J Transl Med. 2016 Jun 8;14:165. doi: 10.1186/s12967-016-0921-x (PMC4898408; doi:10.1186/s12967-016-0921-x)
Supplement: Supplementary file 1 — 10.1186/s12967-016-0921-x Integrated analysis of network pharmacology-based prediction and experimental validation reveals that Guizhi-Shaoyao-Zhimu decoction attenuates rheumatoid arthritis partially by reversing inflammation-immune system imbalance. [file 12967_2016_921_MOESM1_ESM.pdf]

Supplementary File S1 for " Integrated Analysis of Network Pharmacology-Based Prediction and Experimental Validation Reveals that Guizhi-Shaoyao-Zhimu Decoction Attenuates Rheumatoid Arthritis Partially by Reversing Inflammation-Immune System Imbalance "

Authors: Qiuyan Guo [1#], Xia Mao [1#], Yanqiong ZHANG [1*], Shuqin Meng [2], Yue Xi [2], Yi Ding [2], Xiaocun Zhang [3], Yuntao Dai [1], Xia Liu [3], Chao Wang [1], Yuting Li [1], Na LIN [1*]

List of supplementary files:

File S1:

Section 1. Known RA-related targets

Section 2. Defining network topological feature set

Section 3. Severity assessment of arthritis

Section 4. Histological observation

Table S1 Detailed information on these compositive compounds of each herb in GSZD

Table S2 Known RA-related targets

Table S3 Detailed information on eight existing protein-protein interaction databases

Table S4 Detailed information on the water consumption (mL) of each group throughout 21 day experiment period

**Table S5 Detailed information on the food consumption (g) of each group

throughout 21 day experiment period

Table S6 Detailed information on the body weight (g) changes of each group throughout 21 day experiment period

Table S7 Information of putative targets of nine herbs containing in Guizhi-Shaoyao-Zhimu decoction

Table S8 Pathway enrichment analysis of GSZD putative targets

Table S9 Direct interactions among the hubs of the network of putative targets of GSZD and known RA-related targets

Table S10 Topological features of 40 major hubs

Table S11 Edge-betweenness value of each interaction in the network of direct interactions among the hubs

File S1:

Section 1. Known RA-related targets

Known RA-related targets were obtained from four existing resources: (1) DrugBank database [1] (http://www.drugbank.ca/, version: 3.0). We only used those drug-target interactions whose drugs are FDA approved for the treatment of RA and whose targets are human genes/proteins. In total, we obtained 58 known RA-related targets. (2) The Online Mendelian Inheritance in Man (OMIM) database [2] (http://www.omim.org/, Last updated: October 31, 2013). We searched the OMIM database with a keyword "rheumatoid arthritis" and found 7 known RA-related targets: CD244, HLA-DR1B, MHC2TA, NFKBIL1, PAD, SLC22A4, and PTPN8. (3) Genetic Association Database (GAD) [3] (http://geneticassociationdb.nih.gov/, Last updated: August 18, 2013). We used a keyword "rheumatoid arthritis" to search the GAD database. In total, we obtained 82 known RA-related targets whose association with RA was shown "Y". (4) Kyoto Encyclopedia of Genes and Genomes (KEGG) Pathway Database [4] (http://www.genome.jp/kegg/, Last updated: October 16, 2012). In total, we obtained 92 known RA-related targets which appear on the RA pathway (KEGG ID: map05323) in the KEGG database. The detailed information on these known therapeutic targets is described in **Supplementary Table S7**. After deleting redundancy, there were 208 known RA-related targets collected in this study.

Section 2. Defining network topological feature set

For each node i in interaction network, we defined four measures for assessing its topological property: (1) 'Degree' is defined as the number of links to node i; (2) 'Node betweenness' is defined as the number of shortest paths between pairs of nodes that run through node i. (3) ‘Closeness’ is defined as the inverse of the farness which is the sum of node i distances to all other nodes. The Closeness centrality can be regarded as a measure of how long it will take to spread information from node i to all other nodes sequentially. Degree, node betweenness and closeness centralities can measure a node’s topological importance in the network. The larger a node’s degree/ node betweenness /closeness centrality is, the more important the node is in the interaction network [5]. (4) K-core analysis is an iterative process in which the nodes are removed from the networks in order of least-connected [6]. The core of maximum order is defined as the main core or the highest k-core of the network. A k-core sub-network of the original network can be generated by recursively deleting vertices from the network whose degree is less than k. This results in a series of sub-networks that gradually reveal the globally central region of the original network. On this basis, 'K value' is used to measure the centrality of node i. (5) For each edge in miRNA-regulated protein interaction network, we calculated its 'Edge Betweenness' to assess the importance of a specific interaction in the network. 'Edge Betweenness' is defined as the frequency of an edge that places on the shortest paths between all pairs of vertices in network [7]. The edges with highest betweenness values are most likely to lie between functional modules.

Section 3. Severity assessment of arthritis

Rats were observed once every day after primary immunization. Arthritis severity was evaluated by arthritis score, arthritis incidence, percentage of arthritic limbs and the time of arthritis first appeared which were performed by two independent, blinded observers. The arthritis score was the total of the scores for all 4 limbs (maximum possible arthritis score 80). Arthritis incidence values are the number positive/total number in group. In addition, the number of arthritic limbs of individual rats were counted and added to represent the number of arthritic limbs in a group. The percentage of arthritic limbs in a group was calculated as following formula:

$$\textit{Percentage of arthritic limbs} = \frac{\textit{Number of arthritic limbs in a group}}{\textit{Number of all limbs in a group}} \times 100\%$$

Moreover, the time of arthritis first appeared refered to the first day of the onset of the clinical symptoms of arthritis observed.

Section 4. Histological observation

Rats were sacrificed by cervical dislocation on day 21 after the onset of arthritis. Both hind limbs including the paws, ankles and knees were collected and fixed immediately in 4% paraformaldehyde for 24 h, decalcified in 50% formic acid for up to 1 week at room temperature, and then embedded in paraffin. Sections from paraffin-embedded tissue (4 μm) were stained with hematoxylin and eosin (H&E) or toluidine blue to assess pathologic changes and to monitor the effects of treatment. The sections were evaluated by two pathologists (Prof. M.S. & X.Y.) who were blinded with regard to the experimental conditions of the animals.

References

[1]. Wishart DS, et al. DrugBank: a knowledgebase for drugs, drug actions and drug targets. *Nucleic Acids Res.* **36**, D901-D906 (2008).

[2]. Hamosh A, et al. Online Mendelian Inheritance in Man (OMIM), a knowledgebase of human genes and genetic disorders. *Nucleic Acids Res.* **33(Database issue)**, D514-7 (2005).

[3]. Becker KG, et al. The genetic association database. *Nat Genet.* **36,** 431-2, (2004).

[4]. Wixon J, et al. The Kyoto encyclopedia of genes and genomes--KEGG. *Yeast.* **17**, 48-55 (2000).

[5]. Wang, Y., et al. Drug target prediction based on the herbs components: the study on the multitargets pharmacological mechanism of qishenkeli acting on the coronary heart disease. *Evid. Based Complement Alternat. Med.* **2012**, 698531 (2012).

[6]. Wuchty, S. & Almaas, E. Evolutionary cores of domain co-occurrence networks. *BMC Evol. Biol.* **5**, 24 (2005)

[7]. Narayanan T, et al. Modularity detection in protein-protein interaction networks. *BMC Res Notes.* **4**, 569 (2011).

Table S1 Detailed information on these compositive compounds of each herb in GSZD

Herbs	Chemical_component
Herba Ephedrae	(-)-Epiafzelechin
Rhizoma Zingiberis Recens	(+)-1,5-Epoxy-nor-ketoguaia-11-ene
Ramulus Cinnamomi	(+-)-2,3-Dihydro-2-(1-methylethenyl)-5-benzofurancarboxylic acid methyl ester
Rhizoma Zingiberis Recens	(+-)-Car-3-ene-2,5-dione
Rhizoma Anemarrhenae	(25R,S)-5Alpha-Spirostane-2alpha,3beta-diol 3-O-[O-beta-D-glucopyranosyl-(1-2)-O-beta-D-glucopyranosyl-(1-4)-beta-D-galactopyranoside]
Herba Ephedrae	(4S,5R)Ephedroxane
Radix Glycytthizae	(E)-1-[2,4-Dihydroxy-3-(3-methyl-2-butenyl)
Raidix Saposhnikoviae	(E)-2-Hexenal
Rhizoma Zingiberis Recens	(E)-2-Nonenal
Rhizoma Zingiberis Recens	(E)-Citral
Rhizoma Zingiberis Recens	1-(4-Hydroxy-3-methoxyphenyl-2-[4-(omega-hydroxypropyl)-2-methoxyphenoxy]-propane-1,3-diol
Herba Ephedrae	1,1,3-Trimethylcyclopentane
Paeonia lactiflora	1,2,3,4,6-Pentagalloylglucose
Rhizoma Zingiberis Recens	10-Dehydrogingerdione
Rhizoma Zingiberis Recens	10-Gingediol
Rhizoma Zingiberis Recens	10-Gingerol
Herba Ephedrae	11-Methoxyhumantenine
Rhizoma Atractylodis Macrocephalae	12(Alpha-Methyl butyryl)-14-acetyl-2E,8Z,10E-atractylentriol
Rhizoma Zingiberis Recens	12,13-Di-acetoxyl-1,4,6,11-eudesmanetetol
Paeonia lactiflora	12-Methyl tetradecanoic acid
Rhizoma Anemarrhenae	12-O-Nicotinoylisolineolone
Radix Aconiti Lateralis Preparata	13-Deoxo-13 alpha-acetyloxy-1-deoxynortaxine I
Rhizoma Atractylodis Macrocephalae	14(Alpha-Methyl butyryl)-2E,8E,10E-atractylentriol
Rhizoma Atractylodis Macrocephalae	14(Alpha-Methyl butyryl)-2E,8Z,10E-atractylentriol
Radix Aconiti Lateralis Preparata	14-Deoxy-11,12-didehydroandrographolide
Herba Ephedrae	16-Triacontanol
Paeonia lactiflora	1-O-Beta-d-glucopyranosyl-8-o-benzoylpaeonisuffrone
Paeonia lactiflora	1-O-Beta-d-glucopyranosylpaeonisuffrone
Raidix Saposhnikoviae	1-Octen-3-ol
Rhizoma Zingiberis Recens	1-Thujone
Raidix Saposhnikoviae	2-(Beta-D-glucopyranosyloxy)-8-hydroxy-1-methoxy-3-methyl-9,10-anthraquinone
Radix Glycytthizae	2,5-Dihydroxymethyl-3,4-dihydroxypyrrolidine
Rhizoma Zingiberis Recens	2,5-Dimethyl-7-hydroxy chromone
Radix Aconiti Lateralis Preparata	2,7-Dideacetyl-2,7-dibenzoyl-taxayunnanine F
Rhizoma Atractylodis Macrocephalae	2Alpha-(alpha-Methylbutyrul)-oxy-5alpha,7belta,9alpha,10belta-tetraacetoxy-4(20),11-taxadiene
Rhizoma Zingiberis Recens	2-Heptanol
Paeonia lactiflora	2-Methoxy-5-(e)-propenyl-phenol-beta-vicianoside
Radix Glycytthizae	2-Methyl-1,3,6-trihydroxyanthraquinone
Rhizoma Zingiberis Recens	2-Methyl-5-acetonyl-7-hydroxychromone
Rhizoma Zingiberis Recens	2-Nonanol
Raidix Saposhnikoviae	2-O-Methyl-D-xylose
Ramulus Cinnamomi	3-(4-Hdroxyphenyl)-trans-propenoic acid-2,3-dihydroxypropyl ester
Radix Glycytthizae	3,4-Dicaffeoyl-5-(3-hydroxy-3-methyl) glutaroyl quinic acid
Rhizoma Zingiberis Recens	3-Caraneol
Radix Glycytthizae	3-Hydroxyglabrol (II)
Raidix Saposhnikoviae	3-Methyl-3-butenone
Raidix Saposhnikoviae	3'-O-Acetylhamaudol
Raidix Saposhnikoviae	3'-O-Angeloylhamaudol
Raidix Saposhnikoviae	3-Octanol
Ramulus Cinnamomi	3-O-p-Hydroxy-trans-cinnamoylmaslinic acid
Herba Ephedrae	4,5',8-Trimethyl psoralen
Rhizoma Zingiberis Recens	4,8,12-Trimethyl tridecanoic acid
Rhizoma Atractylodis Macrocephalae	4-Ethoxycarbonyl-2-quinolone
Rhizoma Zingiberis Recens	4-Ethylresorcinol
Rhizoma Zingiberis Recens	4-Gingerol
Rhizoma Anemarrhenae	4-O-Beta-D-Glucopyranosyl methyl gallate
Paeonia lactiflora	4-O-Galloylalbiflorin
Rhizoma Zingiberis Recens	4'-O-Methylglabridin
Paeonia lactiflora	4-O-Methyl-paeoniflorin
Raidix Saposhnikoviae	4-Pentenyl isothiocyanate
Paeonia lactiflora	4-Phenylbicyclo[2,2,2]octan-1-ol
Ramulus Cinnamomi	5-Cinnamoyl-9-O-acetylphototaxicin I
Radix Aconiti Lateralis Preparata	6-Demethyldesoline
Rhizoma Zingiberis Recens	6-Gingediol
Rhizoma Zingiberis Recens	6-Hydroxy-2-[2-(4'-methoxyphenyl)-ethyl] chromone
Rhizoma Zingiberis Recens	6-Methylgingediol
Herba Ephedrae	7-Demethylsuberosin
Paeonia lactiflora	8-Debenzoylpaeonidanin
Rhizoma Zingiberis Recens	8-Geranyloxy psoralen
Rhizoma Zingiberis Recens	8-Gingediol
Radix Glycytthizae	8-Methoxy-5-O-glucoside flavone
Radix Glycytthizae	8-Methyl-10-hydroxylycoctonine
Rhizoma Zingiberis Recens	9-Ethoxy-aristololactam
Rhizoma Zingiberis Recens	Acetylborneol
Radix Aconiti Lateralis Preparata	Aconitine
Rhizoma Zingiberis Recens	Aframodial
Paeonia lactiflora	Albiflorin
Paeonia lactiflora	Albiflorin
Paeonia lactiflora	Albiflorin#
Rhizoma Zingiberis Recens	Alloaromadendrene
Rhizoma Zingiberis Recens	Alpha-Bergamotene
Rhizoma Zingiberis Recens	Alpha-Cedrene
Raidix Saposhnikoviae	Alpha-Cuparenol
Rhizoma Zingiberis Recens	Alpha-Farnesene
Rhizoma Zingiberis Recens	Alpha-Fenchene
Rhizoma Zingiberis Recens	Alpha-Limonene
Herba Ephedrae	Alpha-Teripineol
Radix Glycytthizae	Alpha-Trihydroxy coprostanic acid
Rhizoma Anemarrhenae	Anemarrhenasaponin-I
Rhizoma Anemarrhenae	Anemarrhenasaponin-Ia
Rhizoma Anemarrhenae	Anemarsaponin B
Rhizoma Anemarrhenae	Anemarsaponin C
Rhizoma Anemarrhenae	Anemarsaponin E
Rhizoma Anemarrhenae	Anemarsaponin F
Rhizoma Anemarrhenae	Anemarsaponin G
Herba Ephedrae	Apigenin
Herba Ephedrae	Apigenin-5-rhamnoside
Rhizoma Atractylodis Macrocephalae	Atracty lentrid
Rhizoma Atractylodis Macrocephalae	Atractylenolide I
Rhizoma Atractylodis Macrocephalae	Atractylon
Rhizoma Zingiberis Recens	Bata-Caryophyllene
Paeonia lactiflora	Benzoic acid
Paeonia lactiflora	Benzoylpaeoniflorin
Rhizoma Zingiberis Recens	Beta-Bisabolene
Raidix Saposhnikoviae	Beta-Bisabolene
Rhizoma Zingiberis Recens	Beta-Curcumene
Rhizoma Zingiberis Recens	Beta-Pinene
Raidix Saposhnikoviae	Beta-Pinene
Paeonia lactiflora	Beta-Sitosterol
Rhizoma Zingiberis Recens	Borneol
Rhizoma Zingiberis Recens	Campherenol
Rhizoma Zingiberis Recens	Caranine
Radix Aconiti Lateralis Preparata	Carnosifloside I
Rhizoma Anemarrhenae	Chrysanthemaxanthin
Herba Ephedrae	Cibarian
Rhizoma Zingiberis Recens	Cinerins
Rhizoma Zingiberis Recens	Cis-belta-Terpineol
Rhizoma Zingiberis Recens	Cis-Methyl isoeugenol
Herba Ephedrae	Cis-p-2-Menthen-1-ol
Rhizoma Zingiberis Recens	Citral
Rhizoma Zingiberis Recens	Citraurin beta
Rhizoma Zingiberis Recens	Citronellyl acetate
Rhizoma Zingiberis Recens	Citropten
Rhizoma Zingiberis Recens	Coprine
Ramulus Cinnamomi	Coumarinic acid
Rhizoma Zingiberis Recens	Cubebin
Rhizoma Zingiberis Recens	Cynanuriculoside A
Rhizoma Zingiberis Recens	D-alpha-Pipecoline
Paeonia lactiflora	Daucosterol
Raidix Saposhnikoviae	Dauricine
Rhizoma Zingiberis Recens	Dehydroglaucine
Radix Aconiti Lateralis Preparata	Delavaconitine
Radix Aconiti Lateralis Preparata	Delbruline
Radix Aconiti Lateralis Preparata	Delbrusine
Radix Aconiti Lateralis Preparata	Delcorine
Radix Aconiti Lateralis Preparata	Delphamine
Radix Aconiti Lateralis Preparata	Delphin
Radix Aconiti Lateralis Preparata	Delsemine B
Radix Aconiti Lateralis Preparata	Delsoline
Rhizoma Zingiberis Recens	Delta7-Menthene
Rhizoma Zingiberis Recens	Delta-Elemene
Radix Aconiti Lateralis Preparata	Deltamine
Rhizoma Zingiberis Recens	Delta-Terpineol
Herba Ephedrae	Delta-Terpineol
Radix Aconiti Lateralis Preparata	Deltoin
Raidix Saposhnikoviae	Deltonin (e)
Radix Aconiti Lateralis Preparata	Demethyldelavaine A
Radix Aconiti Lateralis Preparata	Demethyldelavaine B
Radix Aconiti Lateralis Preparata	Deoxyandrographolide
Rhizoma Anemarrhenae	Desglucolanatigonin II
Rhizoma Zingiberis Recens	Difengpin
Herba Ephedrae	Dimethyl phthalate
Radix Glycytthizae	Dimethyl sebacate

Rhizoma Zingiberis Recens	Elemicin
Rhizoma Zingiberis Recens	Elenolide
Rhizoma Zingiberis Recens	E-Nerolidol
Rhizoma Zingiberis Recens	Ethyl aldehyde
Rhizoma Zingiberis Recens	Ethyllaurate
Rhizoma Zingiberis Recens	Eudesobovatol A
Raidix Saposhnikoviae	Eudesobovatol A
Paeonia lactiflora	Eugeniin
Paeonia lactiflora	Eugenitin
Rhizoma Zingiberis Recens	Eugenone
Raidix Saposhnikoviae	Fallacinol
Rhizoma Zingiberis Recens	Farnesiferol A
Rhizoma Zingiberis Recens	Farnesyl acetate
Rhizoma Zingiberis Recens	Fenfangjine A
Radix Glycytthizae	Formononetin-7-glucoside
Rhizoma Zingiberis Recens	Galanthamine
Paeonia lactiflora	Galloylpaeoniflorin
Paeonia lactiflora	Galuteolin
Rhizoma Zingiberis Recens	Gamma-Camphorene
Radix Glycytthizae	Gamma-Sitosterol
Raidix Saposhnikoviae	Gamma-Sitosterol
Ramulus Cinnamomi	Gamma-Sitosterol
Paeonia lactiflora	Gamma-Sitosterol
Radix Glycytthizae	Gancaonin B
Radix Glycytthizae	Gancaonin C
Radix Glycytthizae	Gancaonin D
Radix Glycytthizae	Gancaonin E
Radix Glycytthizae	Gancaonin F
Radix Glycytthizae	Gancaonin P-3'-methylether
Radix Glycytthizae	Ganoderic acid A
Rhizoma Zingiberis Recens	Geraniin
Rhizoma Zingiberis Recens	Geranyl acetate
Rhizoma Zingiberis Recens	Gingerdione
Rhizoma Zingiberis Recens	Gingerenone A
Rhizoma Zingiberis Recens	Gingerenone B
Rhizoma Zingiberis Recens	Gingerenone C
Rhizoma Zingiberis Recens	Gingerol
Rhizoma Anemarrhenae	Ginkgolic acid
Radix Glycytthizae	Gloeosteretriol
Herba Ephedrae	Glucoraphanin
Radix Glycytthizae	Glycyphyllin
Radix Glycytthizae	Glycyrol
Radix Glycytthizae	Glycyroside
Radix Glycytthizae	Glycyrrhetinic acid
Radix Glycytthizae	Glycyrrhetol
Radix Glycytthizae	Glycyrrhisoflavanone
Radix Glycytthizae	Glycyrrhisoflavone
Radix Glycytthizae	Glycyrrhiza-flavonol A
Radix Glycytthizae	Glycyrrhizic acid
Radix Glycytthizae	Glyeurysaponin
Rhizoma Zingiberis Recens	Glypallichalcone
Radix Glycytthizae	Glyyunnanprosapogenin D
Radix Glycytthizae	Gmelofuran
Raidix Saposhnikoviae	Hancinol
Raidix Saposhnikoviae	Heptadeca-1-en-4,6-dihy-3,9-diol
Raidix Saposhnikoviae	Heptadecane
Herba Ephedrae	Herbacetin-3-beta-D-(2-O-beta-D-glucopyra-nosidogluco-pyranoside)-8-beta-D-glucopyranoside
Raidix Saposhnikoviae	Hexanol
Rhizoma Anemarrhenae	Hippeastrine
Radix Glycytthizae	Hispaglabridin B
Radix Glycytthizae	Hispidulin
Rhizoma Zingiberis Recens	Hptaphylline
Radix Aconiti Lateralis Preparata	Hypaphorine
Rhizoma Zingiberis Recens	I-Linalool
Raidix Saposhnikoviae	Imperialine
Paeonia lactiflora	Isobenzoylpaeoniflorin
Rhizoma Zingiberis Recens	Isobrucine
Rhizoma Zingiberis Recens	Isoginkgetin
Radix Glycytthizae	Isogosferol
Radix Glycytthizae	Isoliensinine
Radix Glycytthizae	Isoliquiritin
Radix Glycytthizae	Isolobelanine
Rhizoma Anemarrhenae	Isomangiferolic acid
Radix Glycytthizae	Isoorientin
Paeonia lactiflora	Isopaeoniflorin
Radix Glycytthizae	Isoramanone
Radix Glycytthizae	Isotrilobine
Rhizoma Zingiberis Recens	Isovaleric acid
Rhizoma Atractylodis Macrocephalae	Jurubine
Herba Ephedrae	Kaempferol-3-arabofuranoside
Herba Ephedrae	Kaempferol-7-rhamnoside
Radix Aconiti Lateralis Preparata	Karanjin
Raidix Saposhnikoviae	Ledol
Radix Glycytthizae	Lensinine
Herba Ephedrae	Leucopelargonidin
Radix Glycytthizae	Licochalcone A
Radix Glycytthizae	Licoflavone
Radix Glycytthizae	Licoisoflavaone
Radix Glycytthizae	Licopyranocoumarin
Radix Glycytthizae	Licoricesaponine A3
Radix Glycytthizae	Licoricesaponine C2
Radix Glycytthizae	Licoricesaponine D3
Radix Glycytthizae	Licoricesaponine F3
Radix Glycytthizae	Licoricesaponine G2
Radix Glycytthizae	Licoricesaponine H2
Radix Glycytthizae	Licoricesaponine J2
Radix Glycytthizae	Licoricesaponine K2
Radix Glycytthizae	Licoricidin
Radix Glycytthizae	Licoricone
Radix Glycytthizae	Liquiritigenin-7-O-beta-D-(3-O-acetyl)-apiofuranosyl-4'-O-beta-D-glucopyranoside
Radix Glycytthizae	Liquiritin
Radix Glycytthizae	Liquoric acid
Rhizoma Zingiberis Recens	L-Isoborneol
Rhizoma Anemarrhenae	Mangiferolic acid
Herba Ephedrae	Maragenin II
Rhizoma Anemarrhenae	Markogenin-3-O-beta-D-glucopyranosyl-(1-2)-beta-D-galactopyranoside
Rhizoma Anemarrhenae	Marmesin
Ramulus Cinnamomi	Melilotocarpan A
Radix Aconiti Lateralis Preparata	Mescaline
Rhizoma Zingiberis Recens	Mesotrihydroxypiperidine
Radix Aconiti Lateralis Preparata	Meta-Aminophenol
Radix Glycytthizae	Methyl 2-hydroxy-3,4--dimethoxy benzoate
Radix Glycytthizae	Methyl 3-O-beta-D-glucopyranosyl polygalacate
Rhizoma Zingiberis Recens	Methyl ally tetraslfide
Rhizoma Zingiberis Recens	Methyl guaia-1(10),11-dien-15-carboxylate
Rhizoma Zingiberis Recens	Methyl hexadecanate
Radix Glycytthizae	Methyl linoleate
Paeonia lactiflora	Methyl,gallate
Radix Glycytthizae	Methyl-24-hydroxyglycyrrhetate
Rhizoma Zingiberis Recens	Methyl-4-O-methylgallate
Herba Ephedrae	Methyl-7-epiganoderate
Radix Glycytthizae	Methylglycyrrhetate
Radix Glycytthizae	Methylglyoxal
Rhizoma Anemarrhenae	Monomethyl lithospermate
Herba Ephedrae	Myricadiol
Raidix Saposhnikoviae	Naphthazarin
Radix Glycytthizae	Narwedine
Rhizoma Zingiberis Recens	n-Butyl allophanate
Rhizoma Anemarrhenae	Neoglucobrassicin
Radix Glycytthizae	Neohancoside A
Radix Glycytthizae	Neoisopulegol
Radix Aconiti Lateralis Preparata	Neokadsuranic acid B
Radix Glycytthizae	Neomatatabiol
Radix Glycytthizae	Neowilforine
Rhizoma Zingiberis Recens	Nereistoxin
Rhizoma Zingiberis Recens	Nervogenic acid
Rhizoma Anemarrhenae	Nicotine
Rhizoma Zingiberis Recens	n-Octane
Raidix Saposhnikoviae	n-Octane
Rhizoma Zingiberis Recens	Nonanoic acid
Herba Ephedrae	Norerythrostachaldine
Herba Ephedrae	Nor-rubrofusarin
Rhizoma Zingiberis Recens	Octanoic acid
Herba Ephedrae	O-Methylptelefolonium
Radix Glycytthizae	Ononitol
Radix Aconiti Lateralis Preparata	Ortho-Aminophenol
Paeonia lactiflora	Oxypaeoniflorin
Paeonia lactiflora	Oxypaeoniflorin#
Paeonia lactiflora	Oxypeucedanin
Paeonia lactiflora	Paeoniflorigenone
Paeonia lactiflora	Paeoniflorin
Paeonia lactiflora	Paeoniflorin
Paeonia lactiflora	Paeoniflorin#
Paeonia lactiflora	Paeonilactone A
Paeonia lactiflora	Paeonin

Paeonia lactiflora	Paeonin,a
Paeonia lactiflora	Paeonin,b
Paeonia lactiflora	Paeonin,c
Paeonia lactiflora	Paeonol
Paeonia lactiflora	Paeonol
Paeonia lactiflora	Paeonolide
Raidix Saposhnikoviae	Panaxytriol
Radix Aconiti Lateralis Preparata	Para-Aminophenol
Rhizoma Zingiberis Recens	Paravallarine
Raidix Saposhnikoviae	Pentanic acid
Rhizoma Zingiberis Recens	Perilla ketone
Radix Glycytthizae	Phebalosin
Rhizoma Zingiberis Recens	Phellatin
Raidix Saposhnikoviae	Phenethyl alcohol
Herba Ephedrae	Phenethyl caffeate
Rhizoma Zingiberis Recens	p-Menth-2-en-7-ol
Raidix Saposhnikoviae	Primetin
Ramulus Cinnamomi	Procurcumenol
Rhizoma Zingiberis Recens	Propapyriogenin A2
Rhizoma Zingiberis Recens	Propionic acid
Ramulus Cinnamomi	Protocatechuic acid-3-glucoside
Herba Ephedrae	Pseudoginsenoside F11
Paeonia lactiflora	Pyrethrin II
Paeonia lactiflora	Pyrethrosin
Radix Glycytthizae	Ruvoside
Rhizoma Zingiberis Recens	Safynol
Rhizoma Zingiberis Recens	Santamarin
Rhizoma Anemarrhenae	Sarsasapogenin 3-O-4-rhamnosyl-sophoroside
Rhizoma Zingiberis Recens	Shyobunone
Rhizoma Anemarrhenae	Smilagenin-3-O[beta-D-glucopyranosyl-(1-2)]-beta-D-mannopyranoside
Rhizoma Anemarrhenae	Smilagenone
Paeonia lactiflora	Sodium,paeoniflorin,sulphonate
Rhizoma Anemarrhenae	Spongesterol
Raidix Saposhnikoviae	Suffruticoside A
Radix Aconiti Lateralis Preparata	Tamarixinol (I)
Rhizoma Zingiberis Recens	Terinen-4-ol
Rhizoma Zingiberis Recens	Terpinyl acetate
Raidix Saposhnikoviae	Tetradecane
Radix Glycytthizae	Tetrahydroharmine
Radix Glycytthizae	Tetrahydropalmatine
Rhizoma Anemarrhenae	Timosaponin A-1
Rhizoma Anemarrhenae	Timosaponin A-III
Rhizoma Anemarrhenae	Tingenone
Herba Ephedrae	Trans-belta-Terpineol
Rhizoma Zingiberis Recens	Tricyclodehydroisohumulone
Radix Glycytthizae	Uralenin
Radix Glycytthizae	Uralenneoside
Radix Glycytthizae	Uralenol
Radix Glycytthizae	Uralenol-3-methylether
Radix Glycytthizae	Uralsaponin A
Radix Glycytthizae	Uralsaponin B
Radix Glycytthizae	Urea
Radix Glycytthizae	Vicianin
Rhizoma Anemarrhenae	Xuelianlacton e
Rhizoma Zingiberis Recens	Zingiberene
Rhizoma Zingiberis Recens	Zingiberone
Rhizoma Zingiberis Recens	Z-Nerolidol
Rhizoma Zingiberis Recens	Zygacine

Table S2 Known RA-related targets

Genesymbol	Source
ACAT1	DrugBank
ACCN2	DrugBank
AHR	DrugBank
ALOX5	DrugBank
ALPPL2	DrugBank
C1QA	DrugBank
C1QB	DrugBank
C1QC	DrugBank
C1R	DrugBank
C1S	DrugBank
CD80	DrugBank
CD86	DrugBank
CHRNA3	DrugBank
CHUK	DrugBank
CLCNKA	DrugBank
DHFR	DrugBank
DHODH	DrugBank
FCGR1A	DrugBank
FCGR2A	DrugBank
FCGR2B	DrugBank
FCGR2C	DrugBank
FCGR3A	DrugBank
FCGR3B	DrugBank
GSTA2	DrugBank
HAL	DrugBank
HARS	DrugBank
HDC	DrugBank
HPRT1	DrugBank
IFNG	DrugBank
IKBKB	DrugBank
IL1B	DrugBank
IL1R1	DrugBank
KCNQ2	DrugBank
KCNQ3	DrugBank
LTA	DrugBank
MMP9	DrugBank
NFKB2	DrugBank
PDPK1	DrugBank
PLA2G1B	DrugBank
PLA2G2A	DrugBank
PLA2G4A	DrugBank
PPARG	DrugBank
PRDX5	DrugBank
PTGIS	DrugBank
PTGS1	DrugBank
PTGS2	DrugBank
PTK2B	DrugBank
RXRA	DrugBank
SCN4A	DrugBank

SLC38A3	DrugBank
SLC7A11	DrugBank
TLR7	DrugBank
TLR9	DrugBank
TNF	DrugBank
TNFRSF1B	DrugBank
UGT1A9	DrugBank
VDR	DrugBank
A2M	GAD
NAT2	GAD
PARP1	GAD
AMPD1	GAD
ATIC	GAD
BLK	GAD
C5	GAD
CD19	GAD
CD40	GAD
CDK6	GAD
CCR5	GAD
CYP11B2	GAD
CYP17A1	GAD
ESR1	GAD
FCGR2A	GAD
FCGR2B	GAD
FCGR3A	GAD
IL1RN	GAD
IL2	GAD
IL3	GAD
IL4	GAD
IL4R	GAD
IL10	GAD
IRF5	GAD
ITGAV	GAD
ITPA	GAD
KIR2DS2	GAD
LTA	GAD
MICA	GAD
MICB	GAD
MIF	GAD
MMP2	GAD
MMP7	GAD
MMP12	GAD
MMP13	GAD
MTHFR	GAD
NOS2A	GAD
PDCD1	GAD
PLAU	GAD
PRKCH	GAD
PRKCQ	GAD
REL	GAD
SAA1	GAD

CCL21	GAD
SLC11A1	GAD
SLC19A1	GAD
SPP1	GAD
STAT4	GAD
TAP2	GAD
TRB@	GAD
TNFAIP3	GAD
TNFRSF1A	GAD
TNFRSF1B	GAD
TRAF1	GAD
TRAF5	GAD
BAT2	GAD
TIMELESS	GAD
SH2D2A	GAD
CCL26	GAD
SALL3	GAD
IGLV8	GAD
EXOC4	GAD
FCRL3	GAD
CCBE1	GAD
BTLA	GAD
OLIG3	GAD
CTLA4	GAD;KEGG_PASSWAY
HLA-DMA	GAD;KEGG_PASSWAY
HLA-DQA1	GAD;KEGG_PASSWAY
HLA-DQB1	GAD;KEGG_PASSWAY
IFNG	GAD;KEGG_PASSWAY
IL1B	GAD;KEGG_PASSWAY
IL6	GAD;KEGG_PASSWAY
IL18	GAD;KEGG_PASSWAY
MMP1	GAD;KEGG_PASSWAY
MMP3	GAD;KEGG_PASSWAY
TNF	GAD;KEGG_PASSWAY
VEGFA	GAD;KEGG_PASSWAY
ACP5	KEGG_PASSWAY
ANGPT1	KEGG_PASSWAY
ATP6V1A	KEGG_PASSWAY
ATP6V1B1	KEGG_PASSWAY
ATP6V1B2	KEGG_PASSWAY
ATP6V0C	KEGG_PASSWAY
ATP6V1C1	KEGG_PASSWAY
ATP6V1E1	KEGG_PASSWAY
ATP6V0B	KEGG_PASSWAY
ATP6V1G2	KEGG_PASSWAY
ATP6V0A1	KEGG_PASSWAY
ATP6AP1	KEGG_PASSWAY
CD28	KEGG_PASSWAY
CD80	KEGG_PASSWAY
CD86	KEGG_PASSWAY
CSF1	KEGG_PASSWAY

CSF2	KEGG_PASSWAY
CTSK	KEGG_PASSWAY
CTSL1	KEGG_PASSWAY
FLT1	KEGG_PASSWAY
FOS	KEGG_PASSWAY
CXCL1	KEGG_PASSWAY
HLA-DMB	KEGG_PASSWAY
HLA-DOA	KEGG_PASSWAY
HLA-DOB	KEGG_PASSWAY
HLA-DPA1	KEGG_PASSWAY
HLA-DPB1	KEGG_PASSWAY
HLA-DQA2	KEGG_PASSWAY
HLA-DRA	KEGG_PASSWAY
HLA-DRB3	KEGG_PASSWAY
HLA-DRB4	KEGG_PASSWAY
HLA-DRB5	KEGG_PASSWAY
ICAM1	KEGG_PASSWAY
IL1A	KEGG_PASSWAY
IL8	KEGG_PASSWAY
IL11	KEGG_PASSWAY
IL15	KEGG_PASSWAY
IL17A	KEGG_PASSWAY
ITGAL	KEGG_PASSWAY
ITGB2	KEGG_PASSWAY
JUN	KEGG_PASSWAY
LTB	KEGG_PASSWAY
PGF	KEGG_PASSWAY
CCL2	KEGG_PASSWAY
CCL3	KEGG_PASSWAY
CCL3L1	KEGG_PASSWAY
CCL5	KEGG_PASSWAY
CCL20	KEGG_PASSWAY
CXCL6	KEGG_PASSWAY
CXCL5	KEGG_PASSWAY
CXCL12	KEGG_PASSWAY
TEK	KEGG_PASSWAY
TGFB1	KEGG_PASSWAY
TGFB2	KEGG_PASSWAY
TGFB3	KEGG_PASSWAY
TLR2	KEGG_PASSWAY
TLR4	KEGG_PASSWAY
VEGFB	KEGG_PASSWAY
TNFSF11	KEGG_PASSWAY
TNFSF13	KEGG_PASSWAY
TNFRSF11A	KEGG_PASSWAY
ATP6V0E1	KEGG_PASSWAY
ATP6V0D1	KEGG_PASSWAY
ATP6V1F	KEGG_PASSWAY
ATP6V1G1	KEGG_PASSWAY
TCIRG1	KEGG_PASSWAY
TNFSF13B	KEGG_PASSWAY

ATP6V0A2	KEGG_PASSWAY
ATP6V0A4	KEGG_PASSWAY
ATP6V1D	KEGG_PASSWAY
IL23A	KEGG_PASSWAY
ATP6V1H	KEGG_PASSWAY
ATP6V1E2	KEGG_PASSWAY
ATP6V1G3	KEGG_PASSWAY
ATP6V0E2	KEGG_PASSWAY
CCL3L3	KEGG_PASSWAY
MHC2TA	OMIM
NFKBIL1	OMIM
CD244	OMIM
SLC22A4	OMIM;GAD
PADI4	OMIM;GAD
PTPN22	OMIM;GAD
HLA-DR1B	OMIM;GAD;KEGG_PASSWAY

Table S3 Detailed information on eight existing protein-protein interaction databases

**Source_name**	Website	Release number/ Last updated
Human Annotated and Predicted Protein Interaction Database (HAPPI)	http://bio.informatics.iupui.edu/HAPPI/	Version 31.2
Reactome	http://www.reactome.org/	Version 37
Online Predicted Human Interaction Database (OPHID)	http://ophid.utoronto.ca	Version 1.71
InAct	www.ebi.ac.uk/intact/	Version 2.0
Human Protein Reference Database (HPRD)	http://www.hprd.org/	Release 8
Molecular INTeraction Database (MINT)	http://mint.bio.uniroma2.it/mint/download.do	Aug-2011
Database of Interacting Proteins (DIP)	http://dip.doe-mbi.ucla.edu/dip/	Jan-2010
PDZBase	http://icb.med.cornell.edu/services/pdz/start	2004

Table S4 Detailed information on the water consumption (mL) of each group throughout 21 day experiment period

Group / Day	0	2	4	6	8	10	12	14	16	18	20
Normal(n=6)	100	100	95	115	120	115	125	120	115	125	120
Model(n=10)	180	170	170	190	170	160	180	170	140	150	120
Low-dosage(n=8)	130	130	135	150	170	160	150	150	135	130	110
Middle-dosage(n=8)	130	145	135	170	145	185	145	135	150	150	135
High-dosage(n=8)	135	130	145	150	170	190	170	150	135	145	135
MTX(n=8)	145	135	135	160	160	185	135	150	160	135	120

Table S5 Detailed information on the food consumption (g) of each group throughout 21 day experiment period

Group / Day	0	2	4	6	8	10	12	14	16	18	20
Normal(n=6)	62.4	67.2	74.4	76.8	82.8	84	85.2	90	92.4	93.6	96
Model(n=10)	100	110	120	116	102	84	78	52	56	48	50
Low-dosage(n=8)	88	83.2	91.2	100.8	102.4	92.8	78.4	68.8	65.6	56	52.8
Middle-dosage(n=8)	78.4	91.2	97.6	102.4	86.4	83.2	88	73.6	60.8	65.6	59.2
High-dosage(n=8)	92.8	86.4	99.2	94.4	91.2	88	75.2	76.8	68.8	62.4	59.2
MTX(n=8)	88	92.8	94.4	99.2	105.6	102.4	81.6	72	65.6	70.4	49.6

Table S6 Detailed information on the body weight changes of each group throughout 21 day experiment period

Normal

Day / No.	0	2	4	6	8	10	12	14	16	18	20
1	224.3	236	246.6	260.9	273.4	278.4	284.4	293.4	297.7	301.9	303.6
2	223.7	241.1	254.8	263.7	289.2	300.2	308.1	325.6	329.5	336.8	346.3
3	246.8	257.4	272.3	280.7	297.2	305.4	309.8	323.6	328.7	329.4	334.7
4	236.8	251.5	266.3	283.7	295.6	304.5	308.1	323.5	332.1	336.2	345.8
5	235.2	248	258	278.2	284.5	291.5	292.4	300.1	307.6	315.2	315.4
6	236.2	249.9	264.8	276.3	292.8	300.5	309.4	316.4	324.8	333.7	337.6
x	233.8333333	247.32	260.47	273.92	288.78	296.75	302.03	313.77	320.07	325.53	330.57
s	8.694519347	7.6554	9.2012	9.3762	8.816	10.248	10.881	13.711	14.046	14.055	17.34

Model

Day / No.	0	2	4	6	8	10	12	14	16	18	20
1	213.8	228.4	239.8	247.5	261.6	270.3	268.6	238.9	232.6	223.9	223.2
2	237.7	238.3	250.5	261.3	273.2	275.5	256.5	241.6	233.2	228.6	222.3
3	227.8	241.8	262.2	281.4	297.6	310.9	322.6	298.9	285.7	267.5	241.9
4	233.9	241.2	255.6	267.9	282.3	289.9	283.6	253.4	248.1	244.2	239.6
5	235.9	247.3	257.5	269.3	281.6	287.3	285.1	245.2	242.5	243.5	236.3
6	231.2	241.6	249.2	258.4	270.6	283.1	275.9	239.5	234.3	224.3	207.1
7	244.1	251.9	260.9	271.5	287.6	293.1	281.6	246.2	243.8	239.6	235.2
8	238	246.6	260.3	274.2	289.3	298.9	296.5	265.2	258.7	240.4	235
9	233.2	214.7	252.8	263.1	274.8	277.3	258	238.8	234.8	232.6	221.9
10	234	244	252.8	267.4	276.2	284.4	280.8	253	246.4	238.7	223
x	232.96	239.58	254.16	266.2	279.48	287.07	280.92	252.07	246.01	238.33	228.55
s	8.024	10.737	6.7405	9.3168	10.402	11.952	19.159	18.46	16.21	12.696	10.796

Low-dosage

Day / No.	0	2	4	6	8	10	12	14	16	18	20
1	235.4	235.2	243	253.2	269.3	270.8	273.5	254.8	249.3	232	230.8
2	228.9	231.5	242.6	253.7	267.3	269.9	265.2	241.6	229.1	229.3	224.2
3	232.9	234.5	247.8	259.4	279.2	271.4	241.6	232.6	233	229.9	227.2
4	234.2	239.4	250.2	262.4	273.2	274.7	262.5	235.9	230.8	219.5	213.9
5	237.8	247.2	258	269.4	288.3	283.8	289.8	252.6	248.7	243.1	237.5
6	232.2	232.1	245.4	261.9	272.3	295.7	288	292.8	283.7	280.4	277.5
7	223.4	234.7	245.8	259.2	271.8	275.8	278.2	256.5	253.5	236.1	232.2
8	214.2	254.7	271.5	280.7	294.6	304.8	297.7	258.9	258.7	246.6	245
x	229.875	238.66	250.54	262.49	277	280.86	274.56	253.21	248.35	239.61	236.04
s	7.701344966	8.1942	9.7934	8.9734	9.7045	12.963	18.056	18.795	18.121	18.517	19.094

Middle-dosage

Day / No.	0	2	4	6	8	10	12	14	16	18	20
1	237.6	246.8	258	262.4	276.8	281.5	269.3	248.8	234.8	220.7	221.8
2	232.8	238.2	256.5	266.9	278.5	289.9	287.2	283.2	278.8	269	258.4
3	221.7	237.8	251.7	269.4	276.2	270.8	261.3	246.5	238.6	228.2	217.5
4	226.8	240	251.6	264.3	270.8	289.9	279.3	274.4	243.2	242.2	227
5	220.4	238.2	259	278.4	291	304	303.9	280.9	272	269	263.2
6	235.3	249.7	261.8	268.4	278.9	294.5	289.5	268.6	251.8	248.8	264.8
7	230.6	238.8	252.7	267.3	266.8	287.4	273.2	254.9	245.8	226.3	223.9
8	245.2	256.2	276	259.3	300.4	296.8	293.2	254.9	255.2	242.6	250.4
x	231.3	243.21	258.41	267.05	279.93	289.35	282.11	264.03	252.53	243.35	240.88
s	8.3054	6.8969	8.0095	5.6758	10.851	10.048	13.953	14.572	15.664	18.442	20.212

High-dosage

Day / No.	0	2	4	6	8	10	12	14	16	18	20
1	215.8	231.2	244.2	251.4	263	280.8	272.8	255.8	245.1	238.6	238
2	247.8	258.6	272.6	285.7	297.9	310.9	307.7	273.8	265.8	261.4	259.2
3	237.6	253.1	266	277.5	287.6	299.5	283.6	271.5	262	248.6	241.8
4	227	240.6	250.8	259.9	276.9	278.6	286.8	276.8	271.3	262.1	252.7
5	235.1	245.2	253.8	260.7	270.6	286.6	277.6	266.6	259.2	249.9	247.3
6	218.5	243.2	246.4	265.3	287.2	288.5	277.2	267.6	255.6	249.6	243.9
7	232.2	250.2	260.6	271.3	280.9	294	278.4	265.2	251.6	227	226.4
8	229.4	234.7	268	278.9	291.3	307	293	281.2	264.5	253	243.4
x	230.425	244.6	257.8	268.84	281.93	293.24	284.64	269.81	259.39	248.78	244.09
s	10.33202373	9.2234	10.543	11.523	11.438	11.812	11.285	7.8384	8.4104	11.554	9.7726

MTX(0.2g/kg)

Day / No.	0	2	4	6	8	10	12	14	16	18	20
1	231.7	241.1	254.7	271.4	285.3	280.8	272.2	264.5	259.1	248.5	240.5
2	224.5	232.3	247.6	263.7	275.1	289.9	275.7	266.4	254.2	249.7	242.6
3	221.6	235.7	251.2	265.4	278.9	291.5	287.6	278.9	269.8	258.3	250.8
4	234.1	245.6	258.1	271.3	283.4	278.6	269.8	262.4	255.6	248.8	241.4
5	219.6	230.1	243.5	255.6	265.8	283.6	279.5	267.3	259.4	250.6	243.9
6	229.4	238.7	249.4	265.8	276.3	288.5	280.3	267.8	258.1	249.5	242.4
7	230.8	242.1	257.6	268.2	283.4	294	288.1	275.5	262.7	254.8	243.5
8	217.9	226.8	240.2	255.1	264.7	280.5	271.5	265.8	257.2	248.2	245.8
x	226.2	236.55	250.29	264.56	276.61	285.93	278.09	268.58	259.51	251.05	243.86
s	6.1011	9.2234	10.543	11.523	11.438	11.812	11.285	7.8384	8.4104	11.554	9.7726

Group / Day	Normal	Model	Low-dosage	Middle-dosage	High-dosage	MTX
0	233.8333333	232.96	229.88	231.3	230.43	226.2
2	247.3166667	239.58	238.66	243.21	244.6	236.55
4	260.4666667	254.16	250.54	258.41	257.8	250.29
6	273.9166667	266.2	262.49	267.05	268.84	264.56
8	288.7833333	279.48	277	279.93	281.93	276.61
10	296.75	287.07	280.86	289.35	293.24	285.93
12	302.0333333	280.92	274.56	282.11	284.64	278.09
14	313.7666667	252.07	253.21	264.03	269.81	268.58
16	320.0666667	246.01	248.35	252.53	259.39	259.51
18	325.5333333	238.33	239.61	243.35	248.78	251.05
20	330.5666667	228.55	236.04	240.88	244.09	243.86
p(vs Normal)		0.0066	0.0039	0.0132	0.0235	0.0106
p(vs Model)			0.8767	0.6195	0.4152	0.7003

Note:1）Model vs Normal ** p＜0.01; 2) Low-dosage vs Normal ** p ＜0.01; 3) Middle-dosage vs Normal * p＜0.05; 4) High-dosage vs Normal * p＜0.05; 5) MTX vs Normal * p＜0.05; 6) GSZD/MTX vs Model all p>0.05

Table S7 Information of putative targets of nine herbs containing in Guizhi Shaoyao Zhimu decoction

Herbs	Target_name	Target_genesymbol
Ramulus_Cinnamomi	4-aminobutyrate_aminotransferase,_mitochondrial	ABAT
Radix_Glycytthizae	4-aminobutyrate_aminotransferase,_mitochondrial	ABAT
Herba_Ephedrae	4-aminobutyrate_aminotransferase,_mitochondrial	ABAT
Rhizoma_Zingiberis_Recens	4-aminobutyrate_aminotransferase,_mitochondrial	ABAT
Rhizoma_Atractylodis_Macrocephalae	4-aminobutyrate_aminotransferase,_mitochondrial	ABAT
Raidix_Saposhnikoviae	4-aminobutyrate_aminotransferase,_mitochondrial	ABAT
Radix_Aconiti_Lateralis_Preparata	4-aminobutyrate_aminotransferase,_mitochondrial	ABAT
Radix_Glycytthizae	Acetylcholinesterase	ACHE
Rhizoma_Zingiberis_Recens	Acetylcholinesterase	ACHE
Herba_Ephedrae	Alpha-1B_adrenergic_receptor	ADRA1B
Ramulus_Cinnamomi	Beta-1_adrenergic_receptor	ADRB1
Radix_Glycytthizae	Beta-1_adrenergic_receptor	ADRB1
Herba_Ephedrae	Beta-1_adrenergic_receptor	ADRB1
Rhizoma_Zingiberis_Recens	Beta-1_adrenergic_receptor	ADRB1
Rhizoma_Atractylodis_Macrocephalae	Beta-1_adrenergic_receptor	ADRB1
Raidix_Saposhnikoviae	Beta-1_adrenergic_receptor	ADRB1
Radix_Aconiti_Lateralis_Preparata	Beta-1_adrenergic_receptor	ADRB1
Radix_Glycytthizae	Beta-2_adrenergic_receptor	ADRB2
Rhizoma_Zingiberis_Recens	Beta-2_adrenergic_receptor	ADRB2
Rhizoma_Zingiberis_Recens	Delta-aminolevulinic_acid_dehydratase	ALAD
Paeonia_lactiflora	Serum_albumin	ALB
Rhizoma_Zingiberis_Recens	Serum_albumin	ALB
Radix_Glycytthizae	Androgen_receptor	AR
Paeonia_lactiflora	Sodium/potassium-transporting_ATPase_alpha-1_chain;sodium/potassium-transporting ATPase subunit alpha-1	ATP1A1
Radix_Glycytthizae	Sodium/potassium-transporting_ATPase_alpha-1_chain	ATP1A1
Herba_Ephedrae	Sodium/potassium-transporting_ATPase_alpha-1_chain	ATP1A1
Rhizoma_Zingiberis_Recens	Sodium/potassium-transporting_ATPase_alpha-1_chain	ATP1A1
Rhizoma_Anemarrhenae	Sodium/potassium-transporting_ATPase_alpha-1_chain	ATP1A1
Radix_Aconiti_Lateralis_Preparata	Sodium/potassium-transporting_ATPase_alpha-1_chain	ATP1A1
Rhizoma_Zingiberis_Recens	Aurora_B_kinase;aurora kinase B	AURKB
Rhizoma_Zingiberis_Recens	Aurora_C;aurora kinase C	AURKC
Rhizoma_Zingiberis_Recens	Voltage-dependent_T-type_calcium_channel_alpha-1G_subunit;voltage-dependent T-type calcium channel subunit alpha-1G	CACNA1G
Rhizoma_Zingiberis_Recens	Cell_division_protein_kinase_1	CDK1
Rhizoma_Zingiberis_Recens	Cell_division_protein_kinase_2	CDK2
Ramulus_Cinnamomi	Muscarinic_acetylcholine_receptor_M1	CHRM1
Radix_Glycytthizae	Muscarinic_acetylcholine_receptor_M1	CHRM1
Herba_Ephedrae	Muscarinic_acetylcholine_receptor_M1	CHRM1
Rhizoma_Zingiberis_Recens	Muscarinic_acetylcholine_receptor_M1	CHRM1
Rhizoma_Atractylodis_Macrocephalae	Muscarinic_acetylcholine_receptor_M1	CHRM1
Rhizoma_Anemarrhenae	Muscarinic_acetylcholine_receptor_M1	CHRM1
Raidix_Saposhnikoviae	Muscarinic_acetylcholine_receptor_M1	CHRM1
Radix_Aconiti_Lateralis_Preparata	Muscarinic_acetylcholine_receptor_M1	CHRM1
Rhizoma_Zingiberis_Recens	Neuronal_acetylcholine_receptor_subunit_alpha-2	CHRNA2
Radix_Glycytthizae	Chloride_channel_protein_2	CLCN2
Radix_Glycytthizae	Catechol-O-methyl-transferase	COMT
Raidix_Saposhnikoviae	Catechol-O-methyl-transferase	COMT
Rhizoma_Anemarrhenae	Cysteinyl_leukotriene_D4_receptor	CYSLTR1
Radix_Glycytthizae	Diglyceride_acyltransferase	DGAT1
Herba_Ephedrae	Diglyceride_acyltransferase	DGAT1
Rhizoma_Zingiberis_Recens	D(2)_dopamine_receptor	DRD2
Radix_Aconiti_Lateralis_Preparata	D(2)_dopamine_receptor	DRD2
Radix_Glycytthizae	Estrogen_receptor	ESR1
Radix_Aconiti_Lateralis_Preparata	Coagulation_factor_Xa;coagulation factor X	F10
Raidix_Saposhnikoviae	Fatty-acid_amide_hydrolase;fatty-acid amide hydrolase 1	FAAH
Ramulus_Cinnamomi	CD32;low affinity immunoglobulin gamma Fc region receptor II-a	FCGR2A
Radix_Glycytthizae	CD32	FCGR2A
Herba_Ephedrae	CD32	FCGR2A
Rhizoma_Zingiberis_Recens	CD32	FCGR2A
Rhizoma_Atractylodis_Macrocephalae	CD32	FCGR2A
Raidix_Saposhnikoviae	CD32	FCGR2A
Radix_Aconiti_Lateralis_Preparata	CD32	FCGR2A
Rhizoma_Zingiberis_Recens	Ferrochelatase;ferrochelatase, mitochondrial	FECH
Paeonia_lactiflora	Fibroblast_growth_factor_receptor_2	FGFR2
Radix_Glycytthizae	Fibroblast_growth_factor_receptor_2	FGFR2
Ramulus_Cinnamomi	Farnesyl_protein_transferase;protein farnesyltransferase/geranylgeranyltransferase type-1 subunit alpha	FNTA
Radix_Glycytthizae	Farnesyl_protein_transferase	FNTA
Herba_Ephedrae	Farnesyl_protein_transferase	FNTA
Rhizoma_Zingiberis_Recens	Farnesyl_protein_transferase	FNTA
Rhizoma_Atractylodis_Macrocephalae	Farnesyl_protein_transferase	FNTA
Raidix_Saposhnikoviae	Farnesyl_protein_transferase	FNTA
Radix_Aconiti_Lateralis_Preparata	Farnesyl_protein_transferase	FNTA
Rhizoma_Zingiberis_Recens	Gamma-aminobutyric_acid_receptor_subunit_alpha-	GABRA1
Ramulus_Cinnamomi	Gamma-aminobutyric_acid_receptor_subunit_alpha-1	GABRA1
Radix_Glycytthizae	Gamma-aminobutyric_acid_receptor_subunit_alpha-1	GABRA1
Herba_Ephedrae	Gamma-aminobutyric_acid_receptor_subunit_alpha-1	GABRA1
Rhizoma_Zingiberis_Recens	Gamma-aminobutyric_acid_receptor_subunit_alpha-1	GABRA1
Rhizoma_Atractylodis_Macrocephalae	Gamma-aminobutyric_acid_receptor_subunit_alpha-1	GABRA1
Raidix_Saposhnikoviae	Gamma-aminobutyric_acid_receptor_subunit_alpha-1	GABRA1
Radix_Aconiti_Lateralis_Preparata	Gamma-aminobutyric_acid_receptor_subunit_alpha-1	GABRA1
Rhizoma_Zingiberis_Recens	member 2	GPR172A
Raidix_Saposhnikoviae	member 2	GPR172A
Radix_Aconiti_Lateralis_Preparata	NMDA 2A	GRIN2A
Paeonia_lactiflora	Hemoglobin;hemoglobin subunit beta	HBB
Rhizoma_Zingiberis_Recens	Histone_deacetylase;histone deacetylase 1	HDAC1
Paeonia_lactiflora	HMG-CoA_reductase	HMGCR
Ramulus_Cinnamomi	Heat_shock_protein_HSP_90;heat shock protein HSP 90-alpha	HSP90AA1
Radix_Glycytthizae	Heat_shock_protein_HSP_90	HSP90AA1
Herba_Ephedrae	Heat_shock_protein_HSP_90	HSP90AA1
Rhizoma_Zingiberis_Recens	Heat_shock_protein_HSP_90	HSP90AA1
Rhizoma_Atractylodis_Macrocephalae	Heat_shock_protein_HSP_90	HSP90AA1
Raidix_Saposhnikoviae	Heat_shock_protein_HSP_90	HSP90AA1
Radix_Aconiti_Lateralis_Preparata	Heat_shock_protein_HSP_90	HSP90AA1
Rhizoma_Zingiberis_Recens	5-hydroxytryptamine_1B_receptor;5-hydroxytryptamine receptor 1B	HTR1B
Rhizoma_Zingiberis_Recens	5-hydroxytryptamine_receptor_3B;5-hydroxytryptamine receptor 1B	HTR1B
Rhizoma_Zingiberis_Recens	5-hydroxytryptamine_1D_receptor;5-hydroxytryptamine receptor 1D	HTR1D
Rhizoma_Zingiberis_Recens	5-hydroxytryptamine_receptor_3A	HTR3A
Rhizoma_Zingiberis_Recens	Indoleamine_2,3-dioxygenase_(IDO)_(EC_1.13.11.52)_(Indoleamine-pyrrole_2,3-dioxygenase);indoleamine 2,3-dioxygenase 1	IDO1
Herba_Ephedrae	Alpha-glucosidase	MGAM
Rhizoma_Anemarrhenae	Alpha-glucosidase	MGAM
Radix_Aconiti_Lateralis_Preparata	Alpha-glucosidase	MGAM
Radix_Glycytthizae	Maltase-glucoamylase,_intestinal	MGAM
Radix_Glycytthizae	Motilin_receptor	MLNR
Herba_Ephedrae	Motilin_receptor	MLNR
Rhizoma_Anemarrhenae	Motilin_receptor	MLNR
Radix_Glycytthizae	Phosphomannose_isomerase;phosphomannose isomerase 1	MPI
Rhizoma_Anemarrhenae	Thrombopoietin_receptor	MPL
Rhizoma_Zingiberis_Recens	Melatonin_receptor_type_1A	MTNR1A
Radix_Glycytthizae	Serine/threonine-protein_kinase_mTOR	MTOR
Radix_Glycytthizae	Alpha-N-acetylglucosaminidase	NAGLU
Radix_Glycytthizae	Nitric_oxide_synthase,_inducible	NOS2
Paeonia_lactiflora	Glucocorticoid_receptor	NR3C1
Radix_Glycytthizae	Glucocorticoid_receptor	NR3C1
Herba_Ephedrae	Glucocorticoid_receptor	NR3C1
Rhizoma_Zingiberis_Recens	Glucocorticoid_receptor	NR3C1
Radix_Glycytthizae	Mineralocorticoid_receptor	NR3C2
Rhizoma_Zingiberis_Recens	Mineralocorticoid_receptor	NR3C2
Radix_Glycytthizae	Delta-type_opioid_receptor	OPRD1
Radix_Aconiti_Lateralis_Preparata	Delta-type_opioid_receptor	OPRD1
Ramulus_Cinnamomi	Mu-type_opioid_receptor	OPRM1
Radix_Glycytthizae	Mu-type_opioid_receptor	OPRM1
Herba_Ephedrae	Mu-type_opioid_receptor	OPRM1
Rhizoma_Zingiberis_Recens	Mu-type_opioid_receptor	OPRM1
Rhizoma_Atractylodis_Macrocephalae	Mu-type_opioid_receptor	OPRM1
Raidix_Saposhnikoviae	Mu-type_opioid_receptor	OPRM1
Radix_Aconiti_Lateralis_Preparata	Mu-type_opioid_receptor	OPRM1
Radix_Glycytthizae	Phospholipase_A2	PLA2G1B
Herba_Ephedrae	Phospholipase_A2	PLA2G1B
Rhizoma_Zingiberis_Recens	Phospholipase_A2	PLA2G1B
Rhizoma_Zingiberis_Recens	Plasmin	PLG
Raidix_Saposhnikoviae	Peptidyl-prolyl_cis-trans_isomerase_A;	PPIAL3
Rhizoma_Zingiberis_Recens	Peptidyl-prolyl_cis-trans_isomerase_A;	PPIAL3
Rhizoma_Zingiberis_Recens	Peptidyl-prolyl_cis-trans_isomerase_B	PPIB
Paeonia_lactiflora	Vitamin_K-dependent_protein_C	PROC
Rhizoma_Zingiberis_Recens	Vitamin_K-dependent_protein_C	PROC
Rhizoma_Anemarrhenae	Prostaglandin_E2_receptor,_EP2_subtype	PTGER2
Rhizoma_Anemarrhenae	Prostaglandin_F2-alpha_receptor	PTGFR
Rhizoma_Anemarrhenae	Prostacyclin_receptor	PTGIR
Rhizoma_Zingiberis_Recens	Prostaglandin_G/H_synthase_1	PTGS1
Raidix_Saposhnikoviae	Prostaglandin_G/H_synthase_1	PTGS1
Rhizoma_Zingiberis_Recens	COX-2;prostaglandin G/H synthase 2	PTGS2
Rhizoma_Zingiberis_Recens	Prostaglandin_G/H_synthase_2	PTGS2
Raidix_Saposhnikoviae	Prostaglandin_G/H_synthase_2	PTGS2
Rhizoma_Zingiberis_Recens	Myophosphorylase	PYGM
Radix_Glycytthizae	Retinoic_acid_receptor_alpha	RARA
Rhizoma_Zingiberis_Recens	Retinoic_acid_receptor_alpha	RARA
Rhizoma_Zingiberis_Recens	Sodium_channel_protein_type_1_subunit_alpha	SCN1A
Paeonia_lactiflora	Sodium_channel_protein_type_5_subunit_alpha	SCN5A
Radix_Aconiti_Lateralis_Preparata	Sodium_channel_protein_type_5_subunit_alpha	SCN5A
Paeonia_lactiflora	Corticosteroid-binding_globulin	SERPINA6

Radix_Glycytthizae	Corticosteroid-binding_globulin	SERPINA6
Herba_Ephedrae	Corticosteroid-binding_globulin	SERPINA6
Rhizoma_Zingiberis_Recens	Corticosteroid-binding_globulin	SERPINA6
Radix_Aconiti_Lateralis_Preparata	Antithrombin-III	SERPINC1
Rhizoma_Atractylodis_Macrocephalae	Solute_carrier_family_12_member_1	SLC12A1
Ramulus_Cinnamomi	Solute_carrier_family_12_member_1	SLC12A1
Paeonia_lactiflora	Solute_carrier_family_12_member_1	SLC12A1
Radix_Glycytthizae	Solute_carrier_family_12_member_1	SLC12A1
Herba_Ephedrae	Solute_carrier_family_12_member_1	SLC12A1
Rhizoma_Zingiberis_Recens	Solute_carrier_family_12_member_1	SLC12A1
Raidix_Saposhnikoviae	Solute_carrier_family_12_member_1	SLC12A1
Radix_Aconiti_Lateralis_Preparata	Solute_carrier_family_12_member_1	SLC12A1
Herba_Ephedrae	Norepinephrine_transporter	SLC6A2
Radix_Glycytthizae	Signal_transducer_and_activator_of_transcription_3	STAT3
Rhizoma_Zingiberis_Recens	Signal_transducer_and_activator_of_transcription_3	STAT3
Radix_Glycytthizae	3	STAT3
Rhizoma_Zingiberis_Recens	3	STAT3
Radix_Aconiti_Lateralis_Preparata	Tyrosine_3-monooxygenase	TH
Herba_Ephedrae	Toll-like_receptor_7	TLR7
Raidix_Saposhnikoviae	Toll-like_receptor_7	TLR7
Radix_Aconiti_Lateralis_Preparata	Toll-like_receptor_7	TLR7
Rhizoma_Zingiberis_Recens	Transient_receptor_potential_cation_channel,_subfamily_M,_member_8;transient receptor potential cation channel subfamily M member 8	TRPM8
Radix_Glycytthizae	Tubulin_beta-2_chain	TUBB2C
Rhizoma_Zingiberis_Recens	Tubulin_beta-2_chain	TUBB2C
Radix_Glycytthizae	Beta_Tubulin;	TUBBP1
Rhizoma_Zingiberis_Recens	Beta_Tubulin;	TUBBP1
Radix_Glycytthizae	Tubulin_beta	TUBBP1
Ramulus_Cinnamomi	Vitamin_D_receptor;vitamin D3 receptor	VDR
Paeonia_lactiflora	Vitamin_D_receptor	VDR
Radix_Glycytthizae	Vitamin_D_receptor	VDR
Herba_Ephedrae	Vitamin_D_receptor	VDR
Rhizoma_Zingiberis_Recens	Vitamin_D_receptor	VDR
Rhizoma_Atractylodis_Macrocephalae	Vitamin_D_receptor	VDR
Raidix_Saposhnikoviae	Vitamin_D_receptor	VDR
Radix_Aconiti_Lateralis_Preparata	Vitamin_D_receptor	VDR
Ramulus_Cinnamomi	Wee1A_kinase	WEE1
Radix_Glycytthizae	Wee1A_kinase	WEE1
Herba_Ephedrae	Wee1A_kinase	WEE1
Rhizoma_Zingiberis_Recens	Wee1A_kinase	WEE1
Rhizoma_Atractylodis_Macrocephalae	Wee1A_kinase	WEE1
Raidix_Saposhnikoviae	Wee1A_kinase	WEE1
Radix_Aconiti_Lateralis_Preparata	Wee1A_kinase	WEE1

Table S8 Pathway enrichment analysis of GSZD putative targets

Pathway_ID	KEGG_name	TragetGene_in_this_pathway	TragetGene_in_all_pathway	All_gene_in_this_pathway	all_gene_in_all_pathway	Pvalue	FDR
hsa04080	Neuroactive ligand-receptor interaction - Homo sapiens (human)	20	62	275	6788	1.18E-11	1.51E-09
hsa04620	Toll-like receptor signaling pathway - Homo sapiens (human)	9	62	200	6788	7.81E-05	0.00499719
hsa04380	Osteoclast differentiation - Homo sapiens (human)	8	62	180	6788	0.00017954	0.00766034
hsa04020	Calcium signaling pathway - Homo sapiens (human)	5	62	114	6788	0.00185196	0.05926266
hsa04610	Complement and coagulation cascades - Homo sapiens (human	2	62	21	6788	0.00222158	0.05687247
hsa04370	VEGF signaling pathway - Homo sapiens (human)	3	62	48	6788	0.00247738	0.05285071

Table S9 Direct interactions among the hubs of the network of putative targets of GSZD and known RA-related targets

NodeA	NodeB
ACAT1	COPS5
ACAT1	SUMO1
ADRA1B	ALB
ADRA1B	CACNA1I
ADRA1B	FN1
ADRA1B	IGHV3-7
ADRA1B	KRT1
ADRA1B	NCOA3
ADRA1B	PRKCA
ADRA1B	SCN5A
ADRB1	GRB2
ADRB2	GRB2
ADRB2	MAPK1
ADRB2	MDM2
ADRB2	OPRD1
ADRB2	PRKCD
ADRB2	SRC
AHR	AR
AHR	CTNNB1
AHR	EP300
AHR	ESR1
AHR	HSP90AA1
AHR	NCOA1
AHR	NCOA2
AHR	NCOR2
AHR	NEDD8
AHR	NRIP1
AHR	RB1
AHR	RELA
AHR	SMARCA4
AHR	SP1
AHR	SRC
AHR	SUMO2
ALB	CACNA1I
ALB	CDK1
ALB	CTNNB1
ALB	FN1
ALB	HSP90AA1
ALB	HSPA1A
ALB	HSPA5
ALB	IGHV3-7
ALB	KRT1
ALB	NCOA3
ALB	SCN5A
AR	AKT1
AR	ATF2
AR	BRCA1
AR	CALM1
AR	CDK1
AR	CREBBP
AR	CTNNB1
AR	EGFR
AR	EP300
AR	ESR1
AR	HDAC1
AR	HSP90AA1
AR	JUN
AR	KAT5
AR	MAPK1
AR	MDM2
AR	MED1
AR	NCOA1
AR	NCOA2
AR	NCOA3
AR	NCOA6
AR	NCOR1
AR	NCOR2
AR	NFKB1
AR	NR3C1
AR	NR3C2
AR	NRIP1
AR	PARP1
AR	POU2F1

AR	PRKDC
AR	RB1
AR	RELA
AR	SMAD3
AR	SP1
AR	SRC
AR	STAT3
AR	SUMO1
AR	TP53
AR	TRIM24
ATP1A1	ATF2
ATP1A1	EGFR
ATP1A1	MAPK1
ATP1A1	MAPK3
ATP1A1	MYC
ATP1A1	PRKCA
ATP1A1	SRC
AURKB	BRCA1
AURKB	CDC5L
AURKB	CDK1
AURKB	FGFR2
AURKB	HDAC1
AURKB	RB1
AURKB	SIRT1
AURKB	SNW1
AURKB	SUMO1
AURKB	SUMO2
AURKB	TP53
C1QA	C1QB
C1QA	C1QC
C1QA	FN1
C1QA	KRT1
C1QB	C1QC
C1QB	RELA
C1QC	FN1
C1QC	KRT1
C1R	C1QA
C1R	C1QB
C1R	CSNK2A1
C1R	EP300
CACNA1I	FN1
CACNA1I	IGHV3-7
CACNA1I	KRT1
CACNA1I	NCOA3
CACNA1I	SCN5A
CDK1	BRCA1
CDK1	CSNK2A1
CDK1	EGFR
CDK1	FYN
CDK1	KAT5
CDK1	MYC
CDK1	PML
CDK1	RB1
CDK1	SIRT1
CDK1	SNW1
CDK1	TOP2A
CDK1	TP53
CDK1	WEE1
CDK2	BRCA1
CDK2	CDC5L
CDK2	EP300
CDK2	ESR1
CDK2	IKBKG
CDK2	NEDD8
CDK2	PML
CDK2	RB1
CDK2	SMAD3
CDK2	TP53
CDK2	WEE1
CHUK	AKT1
CHUK	CALM1
CHUK	COPS5
CHUK	CREBBP
CHUK	CSNK2A1
CHUK	CTNNB1

CHUK	EP300
CHUK	ESR1
CHUK	HSP90AA1
CHUK	HSP90AB1
CHUK	HSPA1A
CHUK	IKBKE
CHUK	IKBKG
CHUK	MYC
CHUK	NCOA2
CHUK	NCOA3
CHUK	NCOR2
CHUK	NEDD8
CHUK	NFKB1
CHUK	NFKB2
CHUK	PRKCA
CHUK	PRKCD
CHUK	PRKDC
CHUK	RELA
CHUK	SMAD3
CHUK	SRC
CHUK	TNF
CHUK	TP53
CHUK	TRAF2
CHUK	TRAF6
CHUK	TUBB
COMT	FN1
DHFR	MDM2
DHFR	RB1
DHFR	SUMO2
DHFR	TP53
DRD2	CALM1
ESR1	AKT1
ESR1	BRCA1
ESR1	CALM1
ESR1	COPS5
ESR1	CREBBP
ESR1	CTNNB1
ESR1	EGFR
ESR1	EP300
ESR1	GRB2
ESR1	HDAC1
ESR1	HSP90AA1
ESR1	HSP90AB1
ESR1	HSPA8
ESR1	JUN
ESR1	KAT5
ESR1	MAPK1
ESR1	MAPK3
ESR1	MDM2
ESR1	MED1
ESR1	NCOA1
ESR1	NCOA2
ESR1	NCOA3
ESR1	NCOA6
ESR1	NCOR1
ESR1	NCOR2
ESR1	NFKB1
ESR1	NRIP1
ESR1	POU2F1
ESR1	RARA
ESR1	RELA
ESR1	SMAD3
ESR1	SMARCA4
ESR1	SNW1
ESR1	SP1
ESR1	SRC
ESR1	TP53
ESR1	TRIM24
ESR1	TUBB
F10	PLG
FCGR2A	FYN
FCGR2B	FYN
FCGR2B	GRB2
FCGR2B	IGHV3-7
FCGR2B	MAPK1

FCGR2B	MAPK3
FCGR2B	SRC
FCGR3A	FCGR1C
FCGR3A	IGHV3-7
FGFR2	FYN
FGFR2	GRB2
FGFR2	IKBKE
FGFR2	IKBKG
FGFR2	TRAF2
FGFR2	TRAF6
GABRA1	PRKCD
HARS	NEDD8
HARS	SUMO1
HARS	TRAF6
HBA1	ATF2
HBA1	HBB
HBB	IGHV3-7
HDAC1	ATF2
HDAC1	BRCA1
HDAC1	CDC5L
HDAC1	CREBBP
HDAC1	CSNK2A1
HDAC1	CTNNB1
HDAC1	EP300
HDAC1	FN1
HDAC1	HDAC2
HDAC1	HSP90AA1
HDAC1	HSP90AB1
HDAC1	HSPA1A
HDAC1	HSPA8
HDAC1	JUN
HDAC1	KAT5
HDAC1	MDM2
HDAC1	MYC
HDAC1	NCOR1
HDAC1	NCOR2
HDAC1	NFKB1
HDAC1	NRIP1
HDAC1	PARP1
HDAC1	PML
HDAC1	POU2F1
HDAC1	RARA
HDAC1	RB1
HDAC1	RELA
HDAC1	SMAD3
HDAC1	SMARCA4
HDAC1	SNW1
HDAC1	SP1
HDAC1	STAT3
HDAC1	SUMO1
HDAC1	SUMO2
HDAC1	TOP2A
HDAC1	TOP2B
HDAC1	TP53
HPRT1	IKBKE
HPRT1	SIRT1
HPRT1	TRAF6
HSP90AA1	AKT1
HSP90AA1	ATF2
HSP90AA1	CALM1
HSP90AA1	CDC5L
HSP90AA1	COPS5
HSP90AA1	CSNK2A1
HSP90AA1	CTNNB1
HSP90AA1	EGFR
HSP90AA1	FYN
HSP90AA1	HDAC2
HSP90AA1	HSP90AB1
HSP90AA1	HSPA1A
HSP90AA1	HSPA5
HSP90AA1	HSPA8
HSP90AA1	IKBKE
HSP90AA1	IKBKG
HSP90AA1	JUN
HSP90AA1	KRT1

HSP90AA1	MAPK1
HSP90AA1	MDM2
HSP90AA1	MYC
HSP90AA1	NEDD8
HSP90AA1	NFKB1
HSP90AA1	NR3C2
HSP90AA1	PRKCA
HSP90AA1	PRKCD
HSP90AA1	PRKDC
HSP90AA1	RELA
HSP90AA1	SIRT1
HSP90AA1	SNW1
HSP90AA1	SP1
HSP90AA1	SRC
HSP90AA1	STAT3
HSP90AA1	SUMO1
HSP90AA1	TP53
HSP90AA1	TRAF2
HSP90AA1	TUBB
IKBKB	AKT1
IKBKB	CALM1
IKBKB	CHUK
IKBKB	COPS5
IKBKB	CREBBP
IKBKB	CTNNB1
IKBKB	HSP90AA1
IKBKB	HSP90AB1
IKBKB	HSPA1A
IKBKB	HSPA5
IKBKB	HSPA8
IKBKB	IKBKE
IKBKB	IKBKG
IKBKB	JUN
IKBKB	NCOA2
IKBKB	NCOA3
IKBKB	NEDD8
IKBKB	NFKB1
IKBKB	NFKB2
IKBKB	PARP1
IKBKB	PRKCA
IKBKB	PRKCD
IKBKB	PRKDC
IKBKB	RELA
IKBKB	SRC
IKBKB	STAT3
IKBKB	TNF
IKBKB	TP53
IKBKB	TRAF2
IKBKB	TRAF6
IKBKB	TUBB
IL1B	ADRB2
IL1B	IL1R1
IL1B	MYC
IL1R1	TRAF6
LTA	TNF
LTA	TNFRSF1B
MMP9	FN1
MMP9	PLG
MTNR1A	HSPA5
MTOR	AKT1
MTOR	PRKCA
MTOR	PRKCD
MTOR	SIRT1
MTOR	STAT3
MTOR	TP53
MTOR	TRAF2
NFKB2	CALM1
NFKB2	CDC5L
NFKB2	HSP90AA1
NFKB2	HSP90AB1
NFKB2	HSPA1A
NFKB2	HSPA5
NFKB2	HSPA8
NFKB2	IKBKE
NFKB2	IKBKG

NFKB2	NFKB1
NFKB2	NR3C1
NFKB2	PARP1
NFKB2	PRKDC
NFKB2	RELA
NFKB2	SMARCA4
NFKB2	SUMO1
NFKB2	TUBB
NOS2	SRC
NR3C1	AKT1
NR3C1	CALM1
NR3C1	CREBBP
NR3C1	EP300
NR3C1	FYN
NR3C1	HDAC1
NR3C1	HDAC2
NR3C1	HSP90AA1
NR3C1	HSPA1A
NR3C1	JUN
NR3C1	KAT5
NR3C1	MAPK1
NR3C1	MDM2
NR3C1	MED1
NR3C1	NCOA1
NR3C1	NCOA2
NR3C1	NCOA6
NR3C1	NCOR1
NR3C1	NCOR2
NR3C1	NFKB1
NR3C1	NR3C2
NR3C1	NRIP1
NR3C1	PML
NR3C1	POU2F1
NR3C1	PRKDC
NR3C1	RARA
NR3C1	RELA
NR3C1	SMAD3
NR3C1	SMARCA4
NR3C1	SRC
NR3C1	STAT3
NR3C1	SUMO1
NR3C1	SUMO2
NR3C1	TP53
NR3C1	TRIM24
NR3C2	TRIM24
OPRD1	OPRM1
OPRD1	PRKCA
OPRD1	PRKCD
OPRM1	CALM1
PDPK1	AKT1
PDPK1	HSP90AA1
PDPK1	IGHV3-7
PDPK1	MTOR
PDPK1	PRKCD
PDPK1	PTK2B
PDPK1	SRC
PLA2G4A	KAT5
PLA2G4A	MYC
PLA2G4A	RELA
PLG	EP300
PLG	FN1
PLG	SMAD3
PPARG	COPS5
PPARG	CREBBP
PPARG	EP300
PPARG	HDAC1
PPARG	HSP90AB1
PPARG	KAT5
PPARG	MAPK1
PPARG	MED1
PPARG	NCOA1
PPARG	NCOA2
PPARG	NCOA3
PPARG	NCOA6
PPARG	NCOR1

PPARG	NCOR2
PPARG	NRIP1
PPARG	PML
PPARG	RB1
PPARG	SCN5A
PPARG	SMARCA4
PPARG	SP1
PPARG	SUMO1
PPARG	TRIM24
PPARG	TUBB
PPIB	ATF2
PPIB	IKBKE
PPIB	TRAF6
PRDX5	ATF2
PRDX5	IKBKE
PTGS2	COPS5
PTGS2	TP53
PTK2B	EGFR
PTK2B	FGFR2
PTK2B	FYN
PTK2B	GRB2
PTK2B	PRKCD
PTK2B	SRC
PTK2B	STAT3
RARA	CREBBP
RARA	EP300
RARA	FN1
RARA	HDAC2
RARA	MAPK1
RARA	MED1
RARA	NCOA1
RARA	NCOA2
RARA	NCOA3
RARA	NCOA6
RARA	NCOR1
RARA	NCOR2
RARA	NRIP1
RARA	PARP1
RARA	PML
RARA	POU2F1
RARA	PRKCA
RARA	PRKCD
RARA	SIRT1
RARA	SNW1
RARA	SP1
RARA	SRC
RARA	SUMO1
RARA	TOP2B
RARA	TP53
RARA	TRIM24
RXRA	CREBBP
RXRA	CTNNB1
RXRA	EP300
RXRA	ESR1
RXRA	HDAC1
RXRA	MAPK1
RXRA	MAPK3
RXRA	MED1
RXRA	NCOA1
RXRA	NCOA2
RXRA	NCOA3
RXRA	NCOA6
RXRA	NCOR1
RXRA	NCOR2
RXRA	NFKB1
RXRA	NR3C2
RXRA	NRIP1
RXRA	PARP1
RXRA	PML
RXRA	POU2F1
RXRA	PPARG
RXRA	RARA
RXRA	RELA
RXRA	SMARCA4
RXRA	SNW1

RXRA	SP1
RXRA	SRC
RXRA	SUMO1
RXRA	TRIM24
SCN5A	CALM1
SCN5A	FN1
SCN5A	HDAC2
SCN5A	IGHV3-7
SCN5A	KRT1
SCN5A	NCOA3
SCN5A	TP53
STAT3	BRCA1
STAT3	CDC5L
STAT3	CREBBP
STAT3	EGFR
STAT3	EP300
STAT3	HDAC2
STAT3	HSP90AB1
STAT3	JUN
STAT3	KAT5
STAT3	MAPK1
STAT3	MAPK3
STAT3	NCOA1
STAT3	NFKB1
STAT3	PML
STAT3	PRKCD
STAT3	RB1
STAT3	RELA
STAT3	SIRT1
STAT3	SMARCA4
STAT3	SP1
STAT3	SRC
STAT3	SUMO2
TH	CSNK2A1
TH	FN1
TH	MAPK1
TH	MAPK3
TH	MYC
TLR9	GRB2
TLR9	HSPA8
TLR9	SRC
TLR9	TRAF6
TNF	CALM1
TNF	HDAC2
TNF	IKBKG
TNF	POU2F1
TNF	TNFRSF1B
TNF	TRAF2
TNFRSF1B	ATP1A1
TNFRSF1B	CALM1
TNFRSF1B	HSPA1A
TNFRSF1B	HSPA5
TNFRSF1B	HSPA8
TNFRSF1B	PRKDC
TNFRSF1B	TRAF2
TNFRSF1B	TRAF6
TNFRSF1B	TUBB
TOP2A	BRCA1
TOP2A	CDC5L
TOP2A	COPS5
TOP2A	CSNK2A1
TOP2A	CTNNB1
TOP2A	HDAC2
TOP2A	JUN
TOP2A	MAPK1
TOP2A	MYC
TOP2A	NEDD8
TOP2A	PARP1
TOP2A	PRKCA
TOP2A	PRKDC
TOP2A	RB1
TOP2A	SUMO1
TOP2A	SUMO2
TOP2A	TOP2B
TOP2A	TP53

TOP2B	HDAC2
TOP2B	MAPK3
TOP2B	NEDD8
TOP2B	PARP1
TOP2B	PML
TOP2B	PRKDC
TOP2B	SMARCA4
TOP2B	SUMO1
TOP2B	SUMO2
TOP2B	TP53
TUBB	ATF2
TUBB	BRCA1
TUBB	CDC5L
TUBB	CTNNB1
TUBB	EGFR
TUBB	HSP90AB1
TUBB	HSPA5
TUBB	HSPA8
TUBB	IKBKE
TUBB	IKBKG
TUBB	JUN
TUBB	KRT1
TUBB	MDM2
TUBB	MED1
TUBB	NCOA6
TUBB	NFKB1
TUBB	RELA
TUBB	SMARCA4
TUBB	SNW1
TUBB	TRAF2
TUBB	TRAF6
VDR	CREBBP
VDR	CSNK2A1
VDR	EP300
VDR	HDAC2
VDR	JUN
VDR	MAPK1
VDR	MAPK3
VDR	MED1
VDR	NCOA1
VDR	NCOA2
VDR	NCOA6
VDR	NCOR1
VDR	NCOR2
VDR	NRIP1
VDR	POU2F1
VDR	RB1
VDR	RXRA
VDR	SIRT1
VDR	SMAD3
VDR	SMARCA4
VDR	SNW1
VDR	SRC
VDR	TOP2B
VDR	TP53
VDR	TRIM24
WEE1	CSNK2A1
WEE1	SMAD3

Table S10 Topological features of 40 major hubs

Major_hubs	Degree	nCloseness	nBetweenness	Coreness
AHR	16	45	0.6021685	9
ALB	12	43.0034142	2.703687429	6
AR	40	53.3898315	5.407869339	9
CALM1	12	44.839859	3.61071682	7
CDK1	16	44.839859	1.885993838	7
CHUK	32	52.0661163	5.816147327	9
COPS5	8	42.5675659	1.141344786	6
CSNK2A1	9	43.4482765	1.295891643	6
EP300	14	44.680851	2.588913918	9
ESR1	43	53.8461533	7.635720253	9
FN1	12	42.1404686	5.528527737	6
HDAC1	43	54.782608	8.575027466	9
HDAC2	10	44.0559425	0.592773378	8
HSP90AA1	47	58.064518	14.62290764	9
HSPA8	8	43.1506844	0.5283584	6
IKBKB	31	50.1992035	4.327487469	9
MAPK1	14	45.4873657	1.723747134	9
MAPK3	8	42.1404686	0.565048337	6
MDM2	8	43.2989693	0.674769461	6
MYC	9	43.0034142	2.091442347	6
NCOA3	11	43.2989693	1.197755575	7
NFKB2	19	45.4873657	0.96369344	9
NR3C1	37	51.4285698	4.638440609	9
PPARG	24	46.4944649	2.32793498	9
PRKCA	9	43.75	1.308900833	6
PRKCD	11	44.2105255	2.779033422	6
RARA	30	49.4117661	4.332801342	9
RB1	11	42.1404686	0.448820025	7
RELA	14	45	2.227921247	9
RXRA	30	47.1910095	1.841027975	9
SIRT1	8	42.7118645	0.884352326	6
SMAD3	9	42.5675659	1.485199332	7
SRC	18	46.4944649	4.293210983	9
STAT3	29	49.6063004	3.416589022	9
SUMO1	13	43.4482765	1.90564096	8
TOP2A	20	43.5986176	1.420408368	8
TOP2B	14	42.2818794	0.512068331	8
TP53	18	46.3235283	3.009473801	9
TNF	28	49.6063004	4.453363419	9
VDR	25	42.2818794	1.693493128	9

Table S11 Edge-betweenness value of each interaction in the network of direct interactions among the hubs

	ACAT1	ADRA1B	ADRB1	ADRB2	AHR	AKT1	ALB	AR	ATF2	ATP1A1	AURKB	BRCA1	C1QA	C1QB	C1QC	C1R	CACNA1I	CALM1	CDC5L	CDK1	CDK2	CHUK	COMT	COPS5	CREBBP	CSNK2A1	CTNNB1	DHFR	DRD2	EGFR	EP300	ESR1	F10	FCGR1C	FCGR2A	FCGR2B	FCGR3A	FGFR2	FN1	FYN	GABRA1	GRB2	HARS	HBA1
ACAT1	0	0	0	0	0	0	0	0	0	0	0	0	0	0	0	0	0	0	0	0	0	0	0	52.6731	0	0	0	0	0	0	0	0	0	0	0	0	0	0	0	0	0	0	0	0
ADRA1B	0	0	0	0	0	0	16.1598	0	0	0	0	0	0	0	0	0	4.52632	0	0	0	0	0	0	0	0	0	0	0	0	0	0	0	0	0	0	0	0	0	34.0481	0	0	0	0	0
ADRB1	0	0	0	0	0	0	0	0	0	0	0	0	0	0	0	0	0	0	0	0	0	0	0	0	0	0	0	0	0	0	0	0	0	0	0	0	0	0	0	0	0	126	0	0
ADRB2	0	0	0	0	0	0	0	0	0	0	0	0	0	0	0	0	0	0	0	0	0	0	0	0	0	0	0	0	0	0	0	0	0	0	0	0	0	0	0	0	0	27.7721	0	0
AHR	0	0	0	0	0	0	0	17.9214	0	0	0	0	0	0	0	0	0	0	0	0	0	0	0	0	0	0	8.15655	0	0	0	14.6853	21.1113	0	0	0	0	0	0	0	0	0	0	0	0
AKT1	0	0	0	0	0	0	0	21.9688	0	0	0	0	0	0	0	0	0	0	0	0	0	18.6797	0	0	0	0	0	0	0	0	0	25.6061	0	0	0	0	0	0	0	0	0	0	0	0
ALB	0	16.1598	0	0	0	0	0	0	0	0	0	0	0	0	0	0	18.7738	0	0	71.0944	0	0	0	0	0	0	25.5995	0	0	0	0	0	0	0	0	0	0	0	56.2132	0	0	0	0	0
AR	0	0	0	0	17.9214	21.9688	0	0	93.1221	0	0	19.7641	0	0	0	0	0	55.3786	0	48.1751	0	0	0	0	12.9584	0	13.582	0	0	23.0295	30.3387	11.8816	0	0	0	0	0	0	0	0	0	0	0	0
ATF2	0	0	0	0	0	0	0	93.1221	0	31.4862785	0	0	0	0	0	0	0	0	0	0	0	0	0	0	0	0	0	0	0	0	0	0	0	0	0	0	0	0	0	0	0	0	0	157.305
ATP1A1	0	0	0	0	0	0	0	0	31.4863	0	0	0	0	0	0	0	0	0	0	0	0	0	0	0	0	0	0	0	0	17.617	0	0	0	0	0	0	0	0	0	0	0	0	0	0
AURKB	0	0	0	0	0	0	0	0	0	0	0	7.95572	0	0	0	0	0	0	9.3446	18.1195	0	0	0	0	0	0	0	0	0	0	0	0	0	0	0	0	0	45.3952	0	0	0	0	0	0
BRCA1	0	0	0	0	0	0	0	19.7641	0	0	7.95572	0	0	0	0	0	0	0	0	14.273	8.97391	0	0	0	0	0	0	0	0	0	0	22.2177	0	0	0	0	0	0	0	0	0	0	0	0
C1QA	0	0	0	0	0	0	0	0	0	0	0	0	0	12.6077	2.07908	19.9335	0	0	0	0	0	0	0	0	0	0	0	0	0	0	0	0	0	0	0	0	0	0	65.0679	0	0	0	0	0
C1QB	0	0	0	0	0	0	0	0	0	0	0	0	12.6077	0	14.4569	11.2085	0	0	0	0	0	0	0	0	0	0	0	0	0	0	0	0	0	0	0	0	0	0	0	0	0	0	0	0
C1QC	0	0	0	0	0	0	0	0	0	0	0	0	2.07908	14.4569	0	0	0	0	0	0	0	0	0	0	0	0	0	0	0	0	0	0	0	0	0	0	0	0	63.9698	0	0	0	0	0
C1R	0	0	0	0	0	0	0	0	0	0	0	0	19.9335	11.2085	0	0	0	0	0	0	0	0	0	0	0	52.5524	0	0	0	0	75.772	0	0	0	0	0	0	0	0	0	0	0	0	0
CACNA1I	0	4.52632	0	0	0	0	18.7738	0	0	0	0	0	0	0	0	0	0	0	0	0	0	0	0	0	0	0	0	0	0	0	0	0	0	0	0	0	0	0	32.1847	0	0	0	0	0
CALM1	0	0	0	0	0	0	0	55.3786	0	0	0	0	0	0	0	0	0	0	0	0	0	38.283	0	0	0	0	0	0	126	0	0	62.7584	0	0	0	0	0	0	0	0	0	0	0	0
CDC5L	0	0	0	0	0	0	0	0	0	0	9.3446	0	0	0	0	0	0	0	0	0	12.3221	0	0	0	0	0	0	0	0	0	0	0	0	0	0	0	0	0	0	0	0	0	0	0
CDK1	0	0	0	0	0	0	71.0944	48.1751	0	0	18.1195	14.273	0	0	0	0	0	0	0	0	0	0	0	0	0	18.0306	0	0	0	17.525	0	0	0	0	0	0	0	0	0	37.9211	0	0	0	0
CDK2	0	0	0	0	0	0	0	0	0	0	0	8.97391	0	0	0	0	0	0	12.3221	0	0	0	0	0	0	0	0	0	0	0	18.0365	60.6005	0	0	0	0	0	0	0	0	0	0	0	0
CHUK	0	0	0	0	0	18.6797	0	0	0	0	0	0	0	0	0	0	0	38.283	0	0	0	0	0	33.3026	17.3018	47.2297	14.3418	0	0	0	53.8878	35.0079	0	0	0	0	0	0	0	0	0	0	0	0
COMT	0	0	0	0	0	0	0	0	0	0	0	0	0	0	0	0	0	0	0	0	0	0	0	0	0	0	0	0	0	0	0	0	0	0	0	0	0	0	126	0	0	0	0	0
COPS5	52.6731	0	0	0	0	0	0	0	0	0	0	0	0	0	0	0	0	0	0	0	0	33.3026	0	0	0	0	0	0	0	0	0	44.44	0	0	0	0	0	0	0	0	0	0	0	0
CREBBP	0	0	0	0	0	0	0	12.9584	0	0	0	0	0	0	0	0	0	0	0	0	0	17.3018	0	0	0	0	0	0	0	0	0	14.1275	0	0	0	0	0	0	0	0	0	0	0	0
CSNK2A1	0	0	0	0	0	0	0	0	0	0	0	0	0	0	0	52.5524	0	0	0	18.0306	0	47.2297	0	0	0	0	0	0	0	0	0	0	0	0	0	0	0	0	0	0	0	0	0	0
CTNNB1	0	0	0	0	8.15655	0	25.5995	13.582	0	0	0	0	0	0	0	0	0	0	0	0	0	14.3418	0	0	0	0	0	0	0	0	0	16.6504	0	0	0	0	0	0	0	0	0	0	0	0
DHFR	0	0	0	0	0	0	0	0	0	0	0	0	0	0	0	0	0	0	0	0	0	0	0	0	0	0	0	0	0	0	0	0	0	0	0	0	0	0	0	0	0	0	0	0
DRD2	0	0	0	0	0	0	0	0	0	0	0	0	0	0	0	0	0	126	0	0	0	0	0	0	0	0	0	0	0	0	0	0	0	0	0	0	0	0	0	0	0	0	0	0
EGFR	0	0	0	0	0	0	0	23.0295	0	17.6169739	0	0	0	0	0	0	0	0	0	17.525	0	0	0	0	0	0	0	0	0	0	0	26.5557	0	0	0	0	0	0	0	0	0	0	0	0
EP300	0	0	0	0	14.6853	0	0	30.3387	0	0	0	0	0	0	0	75.7721	0	0	0	0	18.0365	53.8878	0	0	0	0	0	0	0	0	0	34.0285	0	0	0	0	0	0	0	0	0	0	0	0
ESR1	0	0	0	0	21.1113	25.6061	0	11.8816	0	0	0	22.2177	0	0	0	0	0	62.7584	0	0	60.6005	35.0079	0	44.44	14.1275	0	16.6504	0	0	26.5557	34.0285	0	0	0	0	0	0	0	0	0	0	183.757	0	0
F10	0	0	0	0	0	0	0	0	0	0	0	0	0	0	0	0	0	0	0	0	0	0	0	0	0	0	0	0	0	0	0	0	0	0	0	0	0	0	0	0	0	0	0	0
FCGR1C	0	0	0	0	0	0	0	0	0	0	0	0	0	0	0	0	0	0	0	0	0	0	0	0	0	0	0	0	0	0	0	0	0	0	0	0	126	0	0	0	0	0	0	0
FCGR2A	0	0	0	0	0	0	0	0	0	0	0	0	0	0	0	0	0	0	0	0	0	0	0	0	0	0	0	0	0	0	0	0	0	0	0	0	0	0	0	126	0	0	0	0
FCGR2B	0	0	0	0	0	0	0	0	0	0	0	0	0	0	0	0	0	0	0	0	0	0	0	0	0	0	0	0	0	0	0	0	0	0	0	0	0	0	0	33.9457	0	33.1191	0	0
FCGR3A	0	0	0	0	0	0	0	0	0	0	0	0	0	0	0	0	0	0	0	0	0	0	0	0	0	0	0	0	0	0	0	0	0	126	0	0	0	0	0	0	0	0	0	0
FGFR2	0	0	0	0	0	0	0	0	0	0	45.3953	0	0	0	0	0	0	0	0	0	0	0	0	0	0	0	0	0	0	0	0	0	0	0	0	0	0	0	0	31.1948	0	38.7643	0	0
FN1	0	34.048	0	0	0	0	56.2132	0	0	0	0	0	65.0679	0	63.9698	0	32.1847	0	0	0	0	0	126	0	0	0	0	0	0	0	0	0	0	0	0	0	0	0	0	0	0	0	0	0
FYN	0	0	0	0	0	0	0	0	0	0	0	0	0	0	0	0	0	0	0	37.9211	0	0	0	0	0	0	0	0	0	0	0	0	0	0	126	33.9457	0	31.1948	0	0	0	0	0	0
GABRA1	0	0	0	0	0	0	0	0	0	0	0	0	0	0	0	0	0	0	0	0	0	0	0	0	0	0	0	0	0	0	0	0	0	0	0	0	0	0	0	0	0	0	0	0
GRB2	0	0	126	27.7722	0	0	0	0	0	0	0	0	0	0	0	0	0	0	0	0	0	0	0	0	0	0	0	0	0	0	0	183.757	0	0	0	33.1191	0	38.7643	0	0	0	0	0	0
HARS	0	0	0	0	0	0	0	0	0	0	0	0	0	0	0	0	0	0	0	0	0	0	0	0	0	0	0	0	0	0	0	0	0	0	0	0	0	0	0	0	0	0	0	0
HBA1	0	0	0	0	0	0	0	0	157.305	0	0	0	0	0	0	0	0	0	0	0	0	0	0	0	0	0	0	0	0	0	0	0	0	0	0	0	0	0	0	0	0	0	0	0
HBB	0	0	0	0	0	0	0	0	0	0	0	0	0	0	0	0	0	0	0	0	0	0	0	0	0	0	0	0	0	0	0	0	0	0	0	0	0	0	0	0	0	0	0	50.3726
HDAC1	0	0	0	0	0	0	0	14.3971	117.695	0	54.1731	21.6872	0	0	0	0	0	0	27.7213	0	0	0	0	0	17.3211	37.6026	17.285	0	0	0	31.9941	26.2628	0	0	0	0	0	0	215.91	0	0	0	0	0
HDAC2	0	0	0	0	0	0	0	0	0	0	0	0	0	0	0	0	0	0	0	0	0	0	0	0	0	0	0	0	0	0	0	0	0	0	0	0	0	0	0	0	0	0	0	0
HPRT1	0	0	0	0	0	0	0	0	0	0	0	0	0	0	0	0	0	0	0	0	0	0	0	0	0	0	0	0	0	0	0	0	0	0	0	0	0	0	0	0	0	0	0	0
HSP90AA1	0	0	0	0	45.5202	28.3298	124.619	30.6776	104.731	0	0	0	0	0	0	0	0	66.1992	37.4719	0	0	20.5795	0	59.9446	0	67.3336	19.3369	0	0	33.8336	0	42.3929	0	0	0	0	0	0	111.451	0	0	0	0	0
HSP90AB1	0	0	0	0	0	0	0	0	0	0	0	0	0	0	0	0	0	0	0	0	0	13.7878	0	0	0	0	0	0	0	0	0	18.3733	0	0	0	0	0	0	0	0	0	0	0	0
HSPA1A	0	0	0	0	0	0	25.5369	0	0	0	0	0	0	0	0	0	0	0	0	0	0	18.0749	0	0	0	0	0	0	0	0	0	0	0	0	0	0	0	0	0	0	0	0	0	0
HSPA5	0	0	0	0	0	0	44.6048	0	0	0	0	0	0	0	0	0	0	0	0	0	0	0	0	0	0	0	0	0	0	0	0	0	0	0	0	0	0	0	0	0	0	0	0	0
HSPA8	0	0	0	0	0	0	0	0	0	0	0	0	0	0	0	0	0	0	0	0	0	0	0	0	0	0	0	0	0	0	0	31.2884	0	0	0	0	0	0	0	0	0	0	0	0
IGHV3-7	0	34.9105	0	0	0	0	125.371	0	0	0	0	0	0	0	0	0	27.3545	0	0	0	0	0	0	0	0	0	0	0	0	0	0	0	0	0	0	74.1151	250	0	0	0	0	0	0	0
IKBKB	0	0	0	0	0	17.2192	0	0	0	0	0	0	0	0	0	0	0	32.6217	0	0	0	9.33751	0	31.9196	18.2851	0	13.8238	0	0	0	0	0	0	0	0	0	0	0	0	0	0	0	0	0
IKBKE	0	0	0	0	0	0	0	0	0	0	0	0	0	0	0	0	0	0	0	0	0	51.4528	0	0	0	0	0	0	0	0	0	0	0	0	0	0	0	30.402	0	0	0	0	0	0
IKBKG	0	0	0	0	0	0	0	0	0	0	0	0	0	0	0	0	0	0	0	0	21.8822	22.7813	0	0	0	0	0	0	0	0	0	0	0	0	0	0	0	24.5283	0	0	0	0	0	0
IL1B	0	0	0	38.5226	0	0	0	0	0	0	0	0	0	0	0	0	0	0	0	0	0	0	0	0	0	0	0	0	0	0	0	0	0	0	0	0	0	0	0	0	0	0	0	0
IL1R1	0	0	0	0	0	0	0	0	0	0	0	0	0	0	0	0	0	0	0	0	0	0	0	0	0	0	0	0	0	0	0	0	0	0	0	0	0	0	0	0	0	0	0	0
JUN	0	0	0	0	0	0	0	12.178	0	0	0	0	0	0	0	0	0	0	0	0	0	0	0	0	0	0	0	0	0	0	0	15.4764	0	0	0	0	0	0	0	0	0	0	0	0
KAT5	0	0	0	0	0	0	0	21.3511	0	0	0	0	0	0	0	0	0	0	0	17.4738	0	0	0	0	0	0	0	0	0	0	0	29.6308	0	0	0	0	0	0	0	0	0	0	0	0
KRT1	0	19.6698	0	0	0	0	14.7541	0	0	0	0	0	62.978	0	61.1625	0	21.3501	0	0	0	0	0	0	0	0	0	0	0	0	0	0	0	0	0	0	0	0	0	0	0	0	0	0	0
LTA	0	0	0	0	0	0	0	0	0	0	0	0	0	0	0	0	0	0	0	0	0	0	0	0	0	0	0	0	0	0	0	0	0	0	0	0	0	0	0	0	0	0	0	0
MAPK1	0	0	0	46.2727	0	0	0	23.9061	0	28.1718311	0	0	0	0	0	0	0	0	0	0	0	0	0	0	0	0	0	0	0	0	0	25.8813	0	0	0	46.6201	0	0	0	0	0	0	0	0
MAPK3	0	0	0	0	0	0	0	0	0	24.2104397	0	0	0	0	0	0	0	0	0	0	0	0	0	0	0	0	0	0	0	0	0	44.7113	0	0	0	29.7963	0	0	0	0	0	0	0	0
